# Metabolic Exchange with Non-Alkane-Consuming Pseudomonas stutzeri SLG510A3-8 Improves *n*-Alkane Biodegradation by the Alkane Degrader *Dietzia* sp. Strain DQ12-45-1b

**DOI:** 10.1128/AEM.02931-19

**Published:** 2020-04-01

**Authors:** Bing Hu, Miaoxiao Wang, Shuang Geng, Liqun Wen, Mengdi Wu, Yong Nie, Yue-Qin Tang, Xiao-Lei Wu

**Affiliations:** a Institute for Synthetic Biosystems, Department of Biochemical Engineering, College of Chemistry and Chemical Engineering, Beijing Institute of Technology, Beijing, People’s Republic of China; b Department of Energy and Resource Engineering, College of Engineering, Peking University, Beijing, People’s Republic of China; c School of Pharmacy, China Pharmaceutical University, Nanjing, People’s Republic of China; d Department of Architecture and Environment, Sichuan University, Chengdu, People’s Republic of China; Wageningen University

**Keywords:** flux balance analysis, interspecies metabolite transfer, metabolomics, proteomics, synergistic biodegradation of *n*-alkanes

## Abstract

Biodegradation of alkanes by microbial communities is ubiquitous in nature. Interestingly, the microbial communities with high hydrocarbon-degrading performances are sometimes composed of not only hydrocarbon degraders but also nonconsumers, but the synergistic mechanisms remain unknown. Here, we found that two bacterial strains isolated from Chinese oil fields, *Dietzia* sp. strain DQ12-45-1b and Pseudomonas stutzeri SLG510A3-8, had a synergistic effect on hexadecane (C_16_ compound) biodegradation, even though P. stutzeri could not utilize C_16_ individually. To gain a better understanding of the roles of the alkane nonconsumer P. stutzeri in the C_16_-degrading consortium, we reconstructed a two-species stoichiometric metabolic model, *i*BH1908, and integrated *in silico* prediction with the following *in vitro* validation, a comparative proteomics analysis, and extracellular metabolomic detection. Metabolic interactions between P. stutzeri and *Dietzia* sp. were successfully revealed to have importance in efficient C_16_ degradation. In the process, P. stutzeri survived on C_16_ metabolic intermediates from *Dietzia* sp., including hexadecanoate, 3-hydroxybutanoate, and α-ketoglutarate. In return, P. stutzeri reorganized its metabolic flux distribution to fed back acetate and glutamate to *Dietzia* sp. to enhance its C_16_ degradation efficiency by improving *Dietzia* cell accumulation and by regulating the expression of *Dietzia* succinate dehydrogenase. By using the synergistic microbial consortium of *Dietzia* sp. and P. stutzeri with the addition of the *in silico*-predicted key exchanged metabolites, diesel oil was effectively disposed of in 15 days with a removal fraction of 85.54% ± 6.42%, leaving small amounts of C_15_ to C_20_ isomers. Our finding provides a novel microbial assembling mode for efficient bioremediation or chemical production in the future.

**IMPORTANCE** Many natural and synthetic microbial communities are composed of not only species whose biological properties are consistent with their corresponding communities but also ones whose chemophysical characteristics do not directly contribute to the performance of their communities. Even though the latter species are often essential to the microbial communities, their roles are unclear. Here, by investigation of an artificial two-member microbial consortium in *n*-alkane biodegradation, we showed that the microbial member without the *n*-alkane-degrading capability had a cross-feeding interaction with and metabolic regulation to the leading member for the synergistic *n*-alkane biodegradation. Our study improves the current understanding of microbial interactions. Because “assistant” microbes showed importance in communities in addition to the functional microbes, our findings also suggest a useful “assistant-microbe” principle in the design of microbial communities for either bioremediation or chemical production.

## Abstract (precis)

Many natural and synthetic microbial communities are composed of not only species whose biological properties are consistent with their corresponding communities but also ones whose chemophysical characteristics do not directly contribute to the performance of their communities. Even though the latter species are often essential to the microbial communities, their roles are unclear. Here, by investigation of an artificial two-member microbial consortium in *n*-alkane biodegradation, we showed that the microbial member without the *n*-alkane-degrading capability had a cross-feeding interaction with and metabolic regulation to the leading member for the synergistic *n*-alkane biodegradation. Our study improves the current understanding of microbial interactions. Because “assistant” microbes showed importance in communities in addition to the functional microbes, our findings also suggest a useful “assistant-microbe” principle in the design of microbial communities for either bioremediation or chemical production.

## INTRODUCTION

Bioremediation of petroleum-contaminated environments and microbially enhanced oil recovery are two environment-friendly, cost-effective techniques to solve serious oil-related problems in current society (1, 2). Clearly, these two biotechniques have opposite aims: one for oil removal and the other for oil recovery. However, both of them preferably induce microbes or microbial consortia with the function of *n*-alkane degradation (3–5). Microbial communities are usually more advantageous for hydrocarbon treatment than the individual species by providing a wider substrate spectrum, higher robustness, and better adaptation to the complicated environments (6, 7). Interestingly, it has been observed that outstanding performance of petroleum alkane biodegradation could be achieved by some microbial communities composed of not only alkane consumers but also nonconsumers (8–11). What are the roles of the microbial members who do not appear to contribute to the main function of their communities? First, they might be dormant cells in the microbial communities under current conditions, acting like seeds waiting for suitable environmental conditions (12). Second, the alkane nonconsumers might have some close affiliations (13) or contact-independent interactions (14) with the alkane-degrading members. If the microbial members without the objective function of the microbial communities are active instead of dormant, metabolic exchange is considered to be a key reason for their survival in these habitats, and their activities should have neutral or positive effects on their functional partners possibly through division of labor (15). However, in some cases, either microbial dormancy or labor division patterns are not sufficient to explain some phenomena. For example, Ghazali et al. (16) screened out six bacterial isolates from oil-contaminated soil to construct bacterial consortium 1 (including three isolates) and consortium 2 (including all six isolates). They found that consortium 2 was more effective than consortium 1 for the removal of medium- and long-chain alkanes, especially *n*-tetradecane, in the diesel-contaminated soil, but unexpectedly the three isolates unique to consortium 2 were unable to utilize *n*-tetradecane or its downstream intermediates individually. Zanaroli et al. (17) isolated two microbial consortia from cow manure with remarkable diesel biodegradation activities, especially for the degradation of C_10_ to C_24_ *n*-alkanes. However, except for the fungus Trametes gibbosa, none of the isolated cultivable bacteria grew well on *n*-alkanes or degraded *n*-alkanes individually. Hence, several questions about those microbial communities remain to be answered, such as how the alkane degraders and nonconsumers cooperate with each other to achieve synergistic alkane biodegradation and what the roles of these alkane nonconsumers within the communities are. The newly developed multiple “omics” technologies (18) and mathematical modeling approaches, such as the genome-scale metabolic model (GEM) reconstruction (19), provide promising ways to better understand the microbial communities in nature and bioprocesses.

In this study, we constructed a simple microbial consortium consisting of an alkane-degrading bacterial strain, *Dietzia* sp. strain DQ12-45-1b, and a potential non-alkane-degrading bacterial strain, Pseudomonas stutzeri SLG510A3-8, both of which were isolated from Chinese oil fields, to investigate the communication mechanism in the synthetic microbial consortium via GEM and omics analyses. The genera *Dietzia* and *Pseudomonas* were widely detected in oil fields (20–22). *Dietzia* species are Gram-positive bacteria that are widely distributed in diverse environments, and some *Dietzia* members have been shown to have the potential for hydrocarbon biodegradation (23). Among them, *Dietzia* sp. DQ12-45-1b was one of the few strains that were reportedly able to utilize a broad spectrum of *n*-alkanes (C_6_ to C_40_), and this property was attributed to the collaboration of a CYP153 alkane hydroxylase and an integral membrane alkane monooxygenase-rubredoxin fusion protein, AlkW1, in the strain (24–26). P. stutzeri, a Gram-negative bacterium, was seldom reported to have the capability to degrade hydrocarbons. For instance, P. stutzeri SLG510A3-8 lacked essential alkane degradation genes such as alkane hydroxylase genes (27), indicating its incompetence for growth on *n*-alkanes. However, in line with the fact that P. stutzeri is one of the most ubiquitous and abundant species in hydrocarbon-related environments (28–30), its strains are considered to interact with the hydrocarbon degraders when they are exposed to hydrocarbons and to play important roles in their microbial communities. Here, to investigate the microbial interaction between the hydrocarbon-degrading microbes and nonconsumers when they are exposed to hydrocarbons as the sole carbon source, we reconstructed a two-species GEM of *Dietzia* sp. DQ12-45-1b and P. stutzeri SLG510A3-8 and analyzed the metabolic flux distribution between the two “compartments” in the stoichiometric model using the flux balance analysis (FBA) algorithm. Afterward, we did *in vitro* cultivation, comparative proteomics analysis, and extracellular metabolomics analysis based on the *in silico* prediction of the metabolic exchange between the two strains on hexadecane (C_16_) to understand the mechanism of communication between the two strains. Our finding on the mutualistic interaction between the leading and supporting members reveal a novel assemblage strategy to construct synthetic microbial consortia with high-performing functions.

## RESULTS

### C_16_ degradation by the synthetic microbial consortium.

**FIG 1:**
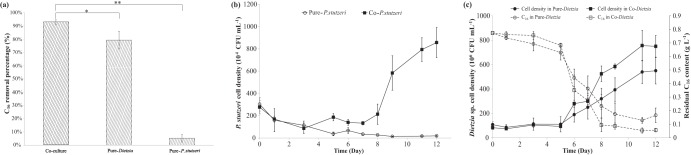
Growth of *Dietzia* sp. DQ12-45-1b and P. stutzeri SLG510A3-8 on hexadecane (C_16_) individually (pure-*Dietzia* sp. and pure-P. stutzeri) or together (co-*Dietzia* sp. and co-P. stutzeri). (a) C_16_ degradation efficiencies, a 12-day growth period, of the microbial community (coculture), and *Dietzia* sp. DQ12-45-1b (pure-*Dietzia* sp.), and P. stutzeri SLG510A3-8 (pure-P. stutzeri). (b) Time courses of P. stutzeri SLG510A3-8 cell densities (CFU ml^−1^) in its monoculture (pure-P. stutzeri) or the coculture system with *Dietzia* sp. DQ12-45-1b (co-P. stutzeri). (c) Time courses of *Dietzia* sp. DQ12-45-1b cell density (CFU ml^−1^) and the residual C_16_ content (g liter^−1^) in its monoculture (pure-*Dietzia*) or the coculture system (co-*Dietzia*). *, *P < *0.05; **, *P < *0.01.

Bacterial strains *Dietzia* sp. strain DQ12-45-1b and P. stutzeri SLG510A3-8 were aerobically cocultivated on C_16_. When P. stutzeri was exposed to C_16_ individually, the percentage of C_16_ removed by P. stutzeri was low in a 12-day culture period (5.21% ± 2.88%) (Fig. 1a), and the P. stutzeri cell density decreased to a very low level (Fig. 1b). This indicated that even if P. stutzeri could adhere to a small amount of C_16_, it was unable to utilize C_16_, which was consistent with the fact that no alkane-oxidizing gene was identified in its genome. For *Dietzia* sp., C_16_ was easily available, since *Dietzia* sp. cells grew well on C_16_ (Fig. 1c), with 79.24% ± 6.71% of the total C_16_ being removed in 12 days in its monoculture (Fig. 1a). Nevertheless, the percentage of C_16_ removal by the microbial consortium (92.94% ± 1.67%) was significantly higher than those by each individual strain (*P < *0.05). On the other hand, both P. stutzeri and *Dietzia* sp. in the coculture system grew much better than those in monocultures, even though the cell density of P. stutzeri was an order of magnitude less than that of *Dietzia* sp. (Fig. 1b and c). By analyzing the time courses of *Dietzia* cell density and residual C_16_ data, it was seen that the specific C_16_ removal rate of *Dietzia* sp. at its highest specific growth rate was significantly higher in the coculture system (1.38 ± 0.10 pg day^−1^ CFU^−1^) than in its monoculture system (0.71 ± 0.16 pg day^−1^ CFU^−1^) (*P < *0.05). Based on the efficient performance in C_16_ degradation by the coculture of P. stutzeri and *Dietzia* sp., a synergistic effect on C_16_ biodegradation through metabolic communication was proposed for the coculture system, in which P. stutzeri might survive on the by-products of *Dietzia* sp. under the C_16_ exposure condition and, in return, secretory metabolites of P. stutzeri are able to induce a higher C_16_ recovery efficiency of the *Dietzia* strain.

### Reconstruction and characterization of the two-species model.

To elucidate the interspecific metabolic communication between P. stutzeri and *Dietzia* sp., two-species metabolic network reconstruction and consortium-level flux balance analysis (FBA) were carried out. First, we reconstructed the single-species GEM of *Dietzia* sp. DQ12-45-1b, namely, *i*BH925 (see File S2 in the supplemental material), and the GEM of P. stutzeri SLG510A3-8, namely, *i*BH983 (see File S3 in the supplemental material). The accuracy of these two models was validated by the consistency between *in vitro* experimental data and *in silico* prediction of growth rates and available carbon source spectra (see File S1 in the supplemental material). Afterward, *i*BH925 and *i*BH983 were reconciled to unify the metabolites and reaction formats, followed by network integration. The resulted model for the microbial consortium, namely, *i*BH1908, contained 1,908 genes, 1,273 metabolites, and 2,670 reactions (see File S4 in the supplemental material). In *i*BH1908, the metabolic networks of *Dietzia* sp. and P. stutzeri were connected to each other through 68 shared metabolites (see Table S1 in File S1). Details of the modeling and analyzing processes of *i*BH925, *i*BH983, and *i*BH1908 are described in File S1.

**FIG 2:**
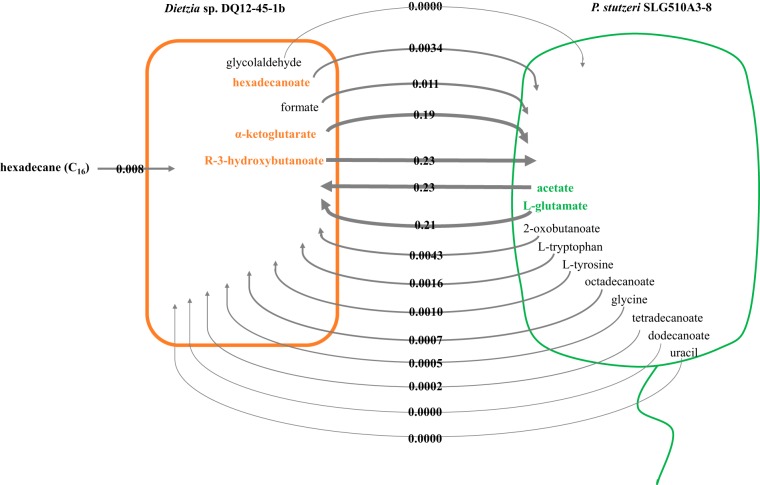
*In silico*-predicted interspecific metabolites that induced the growth of P. stutzeri SLG510A3-8 and *Dietzia* sp. DQ12-45-1b on C_16_ using *i*BH1908.

By using FBA, the flux distribution in *Dietzia* sp. and P. stutzeri was simulated for individual growth or coculture in C_16_-exposed minimal medium. A maximum growth rate μ_0.0718_ = 2.38 × 10^−2^ h^−1^ (see Table S2 in File S1) for *Dietzia* sp. in monoculture was predicted by FBA on *Dietzia* sp. GEM *i*BH925 at the C_16_ uptake rate of 7.18 × 10^−2 ^mmol g^−1^ h^−1^, which was determined by batch culture (see Fig. S1 in File S1). Similarly, with the same nutrient constraints, a maximum growth rate of 0 h^−1^ for P. stutzeri was predicted by FBA on P. stutzeri GEM *i*BH983. In contrast, FBA on the two-species GEM *i*BH1908, in which the *Dietzia* sp. growth rate was constrained to be 2.38 × 10^−2^ h^−1^ while the C_16_ uptake rate was fixed at 8.00 × 10^−2 ^mmol g^−1^ h^−1^ based on our previous measurement (File S1), predicted a maximum growth rate of 5.60 × 10^−3^ h^−1^ for P. stutzeri. Taken together, these predictions were in agreement with our previous findings that no P. stutzeri cell grew on C_16_ individually but its cell density gradually increased in the presence of *Dietzia* sp. (Fig. 1b). The global-level flux distribution in *i*BH1908 reflected that *Dietzia* sp. provided five metabolites to P. stutzeri, while P. stutzeri secreted another 10 compounds for *Dietzia* sp. utilization (Fig. 2). P. stutzeri GEM *i*BH983 and *Dietzia* sp. GEM *i*BH925 were utilized to predict the importance of each individual interspecific metabolite. We simulated the growth rates of P. stutzeri on *i*BH983 via FBA when the absorption rates of the five compounds transferred from *Dietzia* sp. to P. stutzeri, i.e., *R*-3-hydroxybutanoate, α-ketoglutarate, formate, hexadecanoate, and glycolaldehyde, were set at the responding values in Fig. 2. Under these conditions, the maximum predicted growth rate of P. stutzeri increased from 0 to 3.00 × 10^−2^ h^−1^, confirming that the metabolites clearly enhanced P. stutzeri growth. Afterward, the five compounds were taken out one by one in turn, and the growth rates of P. stutzeri were recalculated. The maximum predicted P. stutzeri growth rate (2.99 × 10^−2^ h^−1^) was almost unaffected when formate and glycolaldehyde uptake rates were both constrained to 0 mmol g^−1^ h^−1^. However, the drops were significant when each of the other three compounds was constrained, suggesting that *R*-3-hydroxybutanoate, α-ketoglutarate, and hexadecanoate were key compounds secreted by *Dietzia* sp. to support P. stutzeri growth in the C_16_-exposed minimal medium. Similarly, the 10 P. stutzeri secreted metabolites in Fig. 2 were tested in *i*BH925. Addition of the 10 compounds induced a cell growth rate rise from 2.38 × 10^−2^ to 5.53 × 10^−2^ h^−1^. Acetate and l-glutamate were found to be the key metabolites responsible for the significant rise, as the maximum predicted growth rate of *Dietzia* sp. decreased to 2.56 × 10^−2^ h^−1^ if the two metabolites were excluded.

### *In vitro* validation of *in silico*-predicted key exchanged compounds.

**FIG 3:**
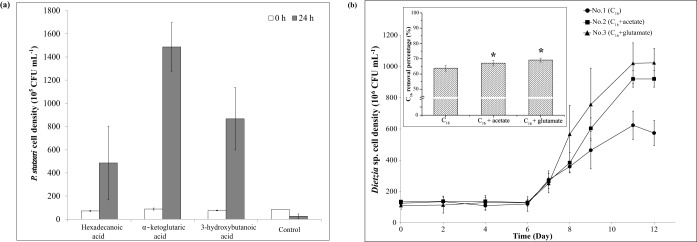
*In vitro* cultivation of P. stutzeri SLG510A3-8 and *Dietzia* sp. DQ12-45-1b on *in silico*-predicted key exchanged compounds. (a) Cell densities of P. stutzeri grown on hexadecanoic acid, α-ketoglutaric acid, *R*-3-hydroxybutyric acid, or no carbon source (control). (b) Time courses of *Dietzia* sp. DQ12-45-1b cell density (graph) and C_16_ removal rates (histogram) in treatments 1, 2, and 3, in which the cultures were supplemented with C_16_ alone, a mixture of C_16_ and acetate, and a mixture of C_16_ and glutamate, respectively. *, *P < *0.05.

Cells of P. stutzeri SLG510A3-8 were inoculated in the minimal medium supplemented with *R*-3-hydroxybutanoic acid, α-ketoglutaric acid, or hexadecanoic acid. As shown in Fig. 3a, P. stutzeri biomass accumulated on each of the three compounds after 24 h of incubation, of which α-ketoglutaric acid was preferred by P. stutzeri more than the other two compounds. The *in vitro* result confirmed that *in silico*-predicted key exchange compounds were able to be utilized by P. stutzeri and suggested that the three compounds might be the reason why P. stutzeri could survive in the coculture system when C_16_ was the sole exogenous carbon source.

In order to test if acetate and glutamate promoted C_16_ biodegradation by *Dietzia* sp. strain DQ12-45-1b, *Dietzia* sp. cells were cultivated in the C_16_-exposed minimal medium with the addition of low concentrations of sodium acetate or sodium glutamate, respectively. Higher *Dietzia* sp. biomass and lower residual C_16_ contents in the cultures supplemented with acetate and glutamate (Fig. 3b) indicated the importance of these two compounds in supporting *Dietzia* sp. growth and C_16_ biodegradation. Furthermore, the specific C_16_ removal rate of *Dietzia* sp. at its highest growth rate in the treatment containing either acetate or glutamate was significantly higher than that in the control (*P < *0.05) (Table 1), which was in agreement with above *in vitro* batch culture result that the specific C_16_ removal rate of *Dietzia* sp. in the coculture system was significantly higher than that in the *Dietzia* sp. monoculture system. At the same time, we tested the effects of the other three compounds, which were *in silico* predicted to be very actively flowing at the interspecific region in *i*BH1908 (*R*-3-hydroxybutanoic acid, α-ketoglutaric acid, and hexadecanoic acid), on *Dietzia* sp. cell growth and C_16_ biodegradation. The specific C_16_ removal rates in *Dietzia* sp. monoculture systems with the addition of these three compounds were not significantly different from those in the monoculture system without any additives (Table 1), which was inconsistent with our *in vitro* data mentioned above. This result indicated that C_16_ biodegradation by *Dietzia* sp. could be enhanced through the *in silico*-predicted P. stutzeri secretions but not by some other compounds.

**TABLE 1:** C_16_ biodegradation kinetics of *Dietzia* sp. DQ12-45-1b in monocultures with C_16_ as the sole carbon source or with the addition of sodium acetate, sodium glutamate, *R*-3-hydroxybutanoic acid, α-ketoglutaric acid, or hexadecanoic acid

Treatment	Carbon source	Highest specific growth rate (day^−1^)^*c*^	*P* value^*a*^	Responding time (days)^*c*^	Responding specific C_16_ removal rate (pg CFU^−1^ day^−1^)^*c*^	*P* value^*b*^
1	C_16_	0.47 ± 0.02		6.99 ± 0.19	0.34 ± 0.04	
2	C_16_ + sodium acetate	0.55 ± 0.03	8.50 × 10^−2^	7.60 ± 0.25	0.54 ± 0.06	1.11 × 10^−2^
3	C_16_ + sodium glutamate	0.76 ± 0.26	0.17	7.19 ± 0.28	0.46 ± 0.05	1.27 × 10^−2^
4	C_16_ + *R*-3-hydroxybutanoic acid	0.47 ± 0.02	0.80	7.35 ± 0.28	0.37 ± 0.03	0.28
5	C_16_ + α-ketoglutaric acid	0.49 ± 0.06	0.52	7.43 ± 0.25	0.35 ± 0.04	9.46 × 10^−2^
6	C_16_ + hexadecanoic acid	0.82 ± 0.12	4.93 × 10^−2^	7.56 ± 0.19	0.37 ± 0.10	0.55

a *P* value between the highest specific growth rates of *Dietzia* sp. DQ12-45-1b in treatment 1 and the responding treatment.

b *P* value between the specific C_16_ removal rates at the times when *Dietzia* sp. DQ12-45-1b had the highest specific growth rates in treatment 1 and the responding treatment.

c Values are ± standard deviation.

### Label-free proteomics and extracellular metabolomics analyses.

**FIG 4:**
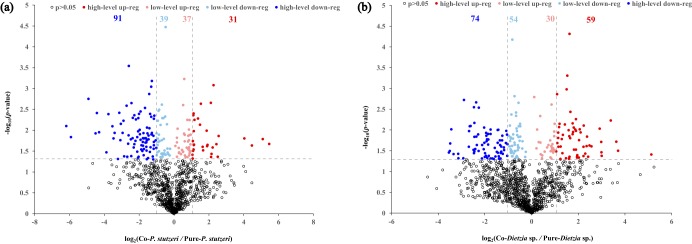
Volcano plot analyses of 1,292 P. stutzeri proteins (a) and 1,541 *Dietzia* sp. proteins (b) identified in coculture triplicates and pure culture triplicates of the two strains by label-free proteomics analysis. In each diagram, the hollowed diamonds below the horizontal dashed line indicate undifferentially expressed proteins in the two treatments (*P > *0.05), and the solid diamonds above the dashed line represent differentially expressed ones (*P < *0.05), of which erythrine represents high-level upregulated proteins (fold change > 2), pale red represents low-level upregulated proteins (1 < fold change < 2), pale blue represents low-level downregulated proteins (0.5 < fold change < 1), and royal blue represents high-level downregulated proteins (fold change < 0.5).

To learn about the metabolic adjustment details of *Dietzia* sp. and P. stutzeri from their monoculture to the coculture states, *Dietzia* sp. and P. stutzeri cells were separately inoculated in each side of a two-chamber bioreactor (see Fig. S2 in File S1), cultivated aerobically on C_16_-exposed minimal medium, and harvested at the mid-exponential phase for proteomics analysis, with the monocultures of each strain as the controls. The results showed that a total of 1,541 *Dietzia* sp. proteins and 1,292 P. stutzeri proteins were detected from trypsin-digested protein mixtures of the coculture and monoculture samples, with a false-discovery rate (FDR) of <0.05. As shown in Fig. 4a, in the case of cocultivation, a total of 68 P. stutzeri proteins were significantly upregulated compared with those in P. stutzeri monoculture system (*P < *0.05), among which 31 proteins showed at least a 2-fold increase in expression values and were termed high-level upregulated proteins, while the remaining 37 proteins were designated low-level upregulated proteins. On the other hand, a total of 91 and 39 P. stutzeri proteins were significantly high-level and low-level downregulated, respectively, in the coculture system (*P < *0.05). Similarly, we found that 59 and 30 *Dietzia* proteins were high- and low-level upregulated in the coculture system, respectively, while 74 and 54 *Dietzia* proteins were high- and low-level downregulated, respectively (Fig. 4b). The functions of the differently expressed proteins for the two strains were identified in NCBI and are listed in Tables S3 and S4 in File S1.

**FIG 5:**
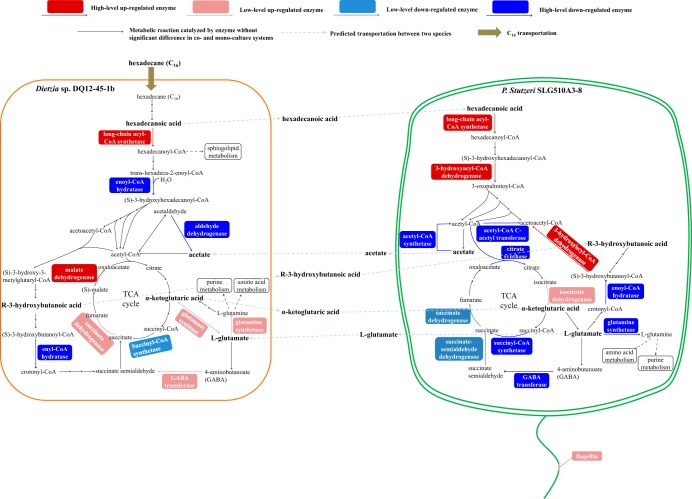
Framework showing the regulation of expression of proteins relating to hexadecanoic acid, 3-hydroxybutanoic acid, α-ketoglutaric acid, acetate, and l-glutamate metabolic pathways in *Dietzia* sp. DQ12-45-1b and P. stutzeri SLG510A3-8 in the artificial consortium when they were exposed to C_16_. C_16_ was consumed by *Dietzia* sp. DQ12-45-1b and was converted to hexadecanoic acid and then to β-oxidation products. Meanwhile, two P. stutzeri enzymes involved in hexadecanoic acid β-oxidation, the long-chain acyl coenzyme A (acyl-CoA) synthetase and the 3-hydroxyacyl-CoA dehydrogenase, had high-level upregulation in the coculture system, indicating that hexadecanoic acid produced by *Dietzia* sp. was assimilated by P. stutzeri in the coculture system. The higher expression of P. stutzeri enzymes involved in hexadecanoic acid β-oxidation and the high-level downexpression of three P. stutzeri enzymes involved in acetate consumption, acetyl-CoA synthetase, acetyl-CoA C-acetyltransferase, and citrate synthase, would lead to the increased production of acetate. Coincidentally, the *Dietzia* sp. enzyme aldehyde dehydrogenase responding for acetate production was high-level downregulated when the cells coexisted with P. stutzeri. The lower expression of this protein could be interpreted as if *Dietzia* sp. indeed utilized extracellular acetate produced by its partner. If this was true, *R*-3-hydroxybutanoate, a downstream product of acetate, might accumulate in *Dietzia* sp. and then be secreted to the surrounding environment for P. stutzeri utilization. This inference was confirmed by the fact that an enoyl-CoA hydratase in P. stutzeri, whose function was the hydrolysis of crotonyl-CoA to the *R*-3-hydroxybutanoate derivative (*S*)-3-hydroxybutanoyl-CoA, was high-level downregulated in the coculture system, while a P. stutzeri 3-hydroxyacyl-CoA dehydrogenase, whose function was the dehydrogenation of (*S*)-3-hydroxybutanoyl-CoA to acetoacetyl-CoA, was high-level upregulated. In addition, if acetate was supplied by P. stutzeri to *Dietzia* sp., α-ketoglutaric acid, a second downstream product of acetate, might accumulate in *Dietzia* sp. and then be converted to l-glutamate in *Dietzia* sp. or be secreted to the surrounding environment for P. stutzeri utilization. This inference was confirmed by the facts that the expression of succinyl-CoA synthetase in *Dietzia*, which used the downstream product of α-ketoglutaric acid as the substrate, was low-level downregulated, while the expression of *Dietzia* glutamate synthase, which catalyzed l-glutamate production from α-ketoglutaric acid, was low-level upregulated. In P. stutzeri, the high-level downregulation of enzymes relating to l-glutamate consumption, such as glutamine synthetase and gamma-aminobutyric acid (GABA) transferase, suggested that l-glutamate might be accumulated and then be secreted to the surrounding environment for *Dietzia* sp. utilization. This hypothesis could explain why the glutamine synthetase and GABA transferase in *Dietzia* sp. were low-level upregulated in the coculture system.

Among the differentially expressed proteins, we focused on proteins relating to the metabolism of C_16_ and the five important interspecific metabolites mentioned above (hexadecanoic acid, α-ketoglutaric acid, *R*-3-hydroxybutanoic acid, acetate, and l-glutamate). Among the differentially expressed proteins, nine proteins in *Dietzia* sp. and 13 proteins in P. stutzeri were involved in the metabolism of the six compounds; the proteins and their fold changes are listed in Tables S3 and S4. As shown in Fig. 5, the up- and downexpression of the 22 enzymes in the microbial consortium could be interpreted as if the five interspecific metabolites indeed transferred between *Dietzia* sp. and P. stutzeri in the *in silico*-predicted directions. Generally, the central metabolism of P. stutzeri became relatively less active in the coculture system than in its monoculture system. In contrast, the tricarboxylic acid (TCA) cycle of *Dietzia* sp. turned out to be more active when the cells were incubated together with P. stutzeri. This indicated that the P. stutzeri cells grown on *Dietzia* sp. secretions might reorganize the flux distribution to produce more acetate and l-glutamate for their partner via stripping their intracellular active metabolism unrelated to acetate and l-glutamate biosynthesis down to essentials while expressing more enzymes relating to acetate and l-glutamate biosynthesis.

In addition, culture samples taken from the two-chamber bioreactors were processed to obtain the cell-free supernatant for determination of the extracellular metabolomic composition. Overall, 43 compounds were detected in the coculture and monoculture systems (see Table S5 in File S1). Among the 43 metabolites, the *in silico*-predicted five key exchanged compounds were all detected in their original or derivatized forms in the supernatant from the coculture system, which was in agreement with above-described proteomic analysis (Fig. 5). Some other *in silico*-predicted interspecific metabolites were also identified in the metabolome profiles, such as dodecanoate, octadecanoate, and tetradecanoate, as well as their derivatized products, which was in agreement with our *in silico* prediction (Table 1). However, the remaining identified extracellular metabolites, such as d-lactic acid, glycolic acid, oxalate, inositol, and the derivatized product of ethanol, were out of the computational prediction. This suggested that the two-species metabolic model could be refined further for a better predictive capability.

### *In silico* prediction of the key *Dietzia* enzyme responding for enhanced C_16_ biodegradation.

According to our proteome data, the relative amounts of the known *Dietzia* alkane hydroxylases AlkW1, AlkW2, and CYP153 showed no significant difference between the monoculture and coculture systems (*P *≥ 0.05) (see Table S6 in File S1), indicating that the synergistic C_16_ biodegradation did not depend on the expression level of *Dietzia* alkane hydroxylases. The key metabolic pathways affecting the C_16_ consumption rate of *Dietzia* sp. in the coculture system should be among the 10 metabolic reactions which were catalyzed by the nine differentially expressed *Dietzia* sp. enzymes (Fig. 5). FBA on *i*BH925 was applied again to determine the key reactions. The model was modified at the nine metabolic reactions and was analyzed using FBA. The modification principle was as follows: first, we obtained the global-level flux distribution of *i*BH925 at its C_16_ uptake rate of 7.18 × 10^−2 ^mmol g^−1^ h^−1^ via FBA (*V*_0.0718_), in which the biomass growth rate μ_0.0718_ (2.38 × 10^−2^ h^−1^) (Table S2) was present; after that, the flux rates of the 10 metabolic reactions mentioned above were constrained to 2-fold (for high-level upregulation), 1.5-fold (for low-level upregulation), 1/1.5 (for low-level downregulation), or 1/2 (for high-level downregulation) of the responding flux values in *V*_0.0718_; next, the C_16_ uptake rate in *i*BH925 was modified to be equal to that in *i*BH1908 (8.00 × 10^−2 ^mmol g^−1^ h^−1^) as mentioned above; and finally, the maximum predicted biomass growth rate μ_0.08_ was computed via FBA again, and μ_0.08_ was compared with μ_0.0718_. If μ_0.08_ with some special reaction flux constraints was similar to μ_0.0718_, the enzymes which catalyzed the special reactions would be considered the key enzymes for the synergistic C_16_ biodegradation in the synthetic microbial consortium. It was found that the value of μ_0.08_ (2.43 × 10^−2^ h^−1^) was similar to that of μ_0.0718_ when the flux rate of Rxn13 catalyzed by succinate dehydrogenase was constrained, but the value was obviously different from that of μ_0.0718_ when the flux rate modification was operated on any one of the remaining nine metabolic reactions. This suggested that the synergistic C_16_ biodegradation in the synthetic microbial consortium might be due to the higher-level expression of succinate dehydrogenase induced by the substrates produced from P. stutzeri. The potential molecular mechanism under which the succinate dehydrogenase positively affects C_16_ biodegradation by *Dietzia* sp. was then examined.

### Enhanced diesel biodegradation by using the synergistic microbial consortium with the addition of *in silico*-predicted key exchanged compounds.

**FIG 6:**
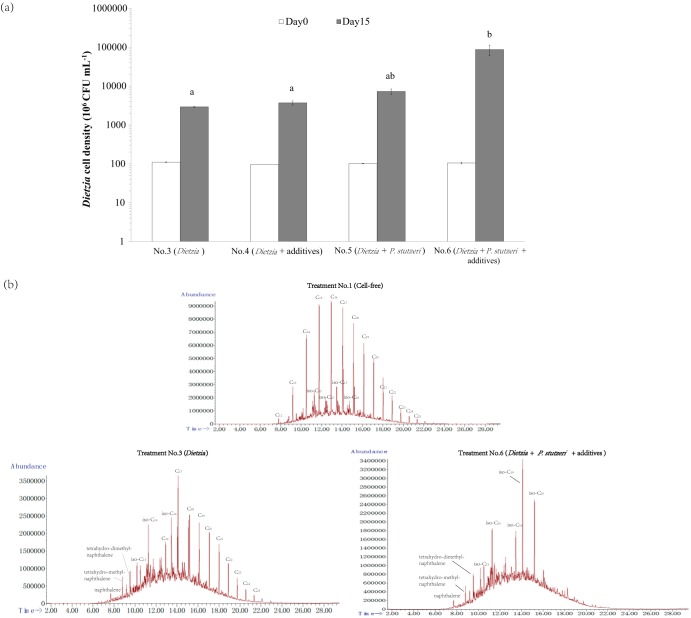
Diesel biodegradation using *Dietzia* sp. DQ12-45-1b, P. stutzeri SLG510A3-8, or their consortium with or without the addition of sodium acetate and sodium glutamate. (a) *Dietzia* cell densities on day 0 (white bars) and day 15 (black bars) in the minimal medium supplemented with 10 g liter^−1^ diesel (treatment 3), with the mixture of sodium acetate and sodium glutamate (treatment 4), with P. stutzeri (treatment 5), and with both the additive mixture and P. stutzeri (treatment 6). Letters above the bars represent significant differences. (b) TIC-GC/MS chromatograms of diesel oil samples after a 15-day disposal in the cell-free culture (treatment 1), treatment 3, and treatment 6.

Accordingly, the synergistic biodegradation capability of the microbial consortium was not limited to C_16_ but also included the synthetic *n*-alkane mixture of C_14_, C_16_, and C_28_ (see Fig. S3 in File S1), and the synergistic effect of the consortium on various *n*-alkanes was proved *in silico* to be enhanced by the sustained supply of acetate and glutamate from P. stutzeri (see Table S7 in File S1). Here, we inferred that the microbial consortium of *Dietzia* sp. and P. stutzeri might have synergistic biodegradation capability on diesel oil, a specific fractional distillate of petroleum composed mainly of alkanes with chain lengths between C_8_ and C_25_ (31), and that acetate and glutamate might be able to improve the microbial consortium-based diesel biodegradation. In order to verify the inferences, *Dietzia* sp. without or with the addition of sodium acetate and sodium glutamate was aerobically cultivated on the minimal medium supplemented with 10 g liter^−1^ diesel oil (treatments 3 and 4, respectively), taking the *Dietzia* sp.-P. stutzeri coculture without or with the additives as two positive controls (treatments 5 and 6, respectively) and cell-free culture and the P. stutzeri monoculture as two negative controls (treatments 1 and 2, respectively). All the cultures were harvested on day 15 for *Dietzia* sp. cell density measurement and determination of the diesel removal percentage. As shown in Fig. 6a, the cell density of *Dietzia* sp. had a significantly higher increase in the coculture systems than in the *Dietzia* sp. monoculture system, especially in treatment 6. As shown in Table 2, P. stutzeri was likely incapable of diesel biodegradation, while *Dietzia* sp. was effective for diesel removal. Notably, the coculture system with the addition of slight amounts of acetate and glutamate had the highest removal percentage (85.54% ± 6.42%), which was over 10% higher than that of the *Dietzia* sp. monoculture (73.53% ± 3.27%). Afterwards, we analyzed the hydrocarbon compositions of the disposed diesel oil and found that the composition profile in the coculture system with additives was much simpler than those in the cell-free culture and *Dietzia* sp. monoculture (Fig. 6b), as well as those in P. stutzeri monoculture and *Dietzia* sp. monoculture with additives (see Fig. S4 in File S1). According to the composition profiles, *n*-alkanes and the isomers with chain lengths between C_12_ and C_14_ were completely removed from the diesel samples in treatments 3 and 6, suggesting a very effective removal of the medium-chain-length *n*-alkanes and their isomers by *Dietzia* sp.; for the *n*-alkanes with chain lengths between C_15_ and C_25_, they were partially removed from the diesel sample in treatment 3 but were completely disposed of in treatment 6, leaving small amounts of C_15_ to C_20_ isomer residues. Our results suggested that the strategy using the synergistic microbial consortium plus *in silico*-predicted interspecific regulators was very efficient for diesel disposal.

**TABLE 2:** Diesel removal in treatments 2 to 6 after a 15-day disposal

Treatment	Bacterium and additives	Diesel removal (%)^*a*^
2	P. stutzeri	1.82 ± 0.09
3	*Dietzia* sp.	73.53 ± 3.27
4	*Dietzia* sp. + additives	76.37 ± 4.13
5	*Dietzia* sp. + P. stutzeri	83.56 ± 5.66
6	*Dietzia* sp. + P. stutzeri + additives	85.54 ± 6.42

a Values are ± standard deviation.

## DISCUSSION

It has been reported that the fraction of hydrocarbon-degrading microorganisms is as high as 1% to 10% of the total population in hydrocarbon-polluted environments, which was usually less than 0.1% in unpolluted environments (32, 33). However, the functions of the remaining 90% to 99% of the microbial populations in these hydrocarbon-related ecosystems were unusually ignored. To reveal a potential contribution of these remaining microbial populations, a microbial consortium consisting of an alkane degrader, *Dietzia* sp. DQ12-45-1b, and a potential alkane nonconsumer, P. stutzeri SLG510A3-8, was investigated. Surprisingly, the microbial consortium was found to have a greater C_16_ removal rate than *Dietzia* sp. alone. The synergistic effect was not limited only to C_16_ degradation but also included degradation of synthetic and natural *n*-alkane mixtures. This finding was in agreement with cases of bioaugmentation with fungal-bacterial consortia, in which the bacterial members acted as the pollutant degraders while the fungal members did not (34, 35). However, the synergistic interaction between our two bacterial strains should be different from that in these cases, since fungi were referred to as the physical vectors for bacterial transport in these consortia because of use of their hyphae. Accordingly, *Dietzia* sp. was realized to convert C_16_ into not only its biomass components but also some metabolic by-products available to P. stutzeri. In return, P. stutzeri fed back some other organic compounds to support the further growth of *Dietzia* sp. and to regulate the expression of some *Dietzia* enzymes, such as succinate dehydrogenase, for improving its C_16_ uptake.

Succinate dehydrogenase catalyzes the electron transfer from succinate to quinone as shown in the following chemical reaction: succinate + quinone → fumarate + quinol. Succinate dehydrogenase is a central metabolic enzyme coupling the bacterial growth process controlled by the TCA cycle with the energy production resulting from the aerobic respiratory chain. Mammalian cells with succinate dehydrogenase deficiency are diagnosed to have syndromic disease (36). For microbials, there are many reports showing that this enzyme is effective for microbial adaptation under different conditions. Hartman et al. reported that succinate dehydrogenase was a governor of Mycobacterium tuberculosis cellular respiration in the adaptation to low-oxygen environments (37). Kiefler et al. significantly increased the carbon conversion efficiency of the industrial obligate aerobic bacterium Gluconobacter oxydans from its cytoplasmic oxidized compounds to biomass by knocking in a heterologous gene cluster expressing succinate dehydrogenase and the responding flavinylation factor (38). Considering that petroleum hydrocarbons cause oxidative stress to bacteria during their biodegradation (5), it is inferred that the upregulation of *Dietzia* sp. succinate reductase induced by the P. stutzeri secretion was used for the oxidative stress adaptation of *Dietzia* sp. during its C_16_ biodegradation, and then the degradation rates in the adaptive *Dietzia* sp. were enhanced. Further studies using molecular techniques on *Dietzia* sp. DQ12-45-1b will show whether succinate dehydrogenase regulates C_16_ biodegradation through oxidative stress adaptation.

Deeply understanding the metabolic interactions among members in microbial consortia with special functions helps to develop efficient microbial consortia or communities in industrial applications. Until now, almost all success stories about the rational design of microbial communities for efficient microbiological processes are based on the following two strategies. The first one is the aggregation method, in which every member could accomplish the tasks of its synthetic microbial consortium but with underperforming efficiencies (39, 40). The second strategy is the “division-of-labor” approach, in which the individual constituents perform complementary roles (41–43). Our study provides a new strategy: the boosting method, in which some members without the desired biological functions promote positive performances of other members who have the desired biological functions or stepwise functions. The microbiological functions of the communities consisting of the supporting and the leading members could be even further enhanced by strengthening their interactions. Taking the consortium of *Dietzia* sp. and P. stutzeri as an example, although its synergistic rate of biodegradation of diesel oil was as high as 83.56% ± 5.66%, we found that the cleanup percentage could be further improved by adding slight amounts of two exogenous compounds closely related to the synergistic *n*-alkane biodegradation (Table 2; see Fig. S3 in File S1 in the supplemental material).

In summary, in our study *Dietzia* sp. DQ12-45-1b and P. stutzeri SLG510A3-8 were found to have a synergistic effect on C_16_ biodegradation, even though P. stutzeri could not assimilate C_16_. By integrating *in silico* prediction and *in vitro* validation, it was found that *Dietzia* sp. and P. stutzeri had a cross-feeding interaction when they were coexposed to C_16_, in which *Dietzia* sp. was the nutrient supplier of P. stutzeri while P. stutzeri was the *n*-alkane degradation regulator donator to *Dietzia* sp. Our finding of the synergy mechanism between the two species provides a novel microbial community assemblage strategy for enhancing functions of microbiological processes and helps to deeply understand microbial behaviors in natural ecosystems in the future.

## MATERIALS AND METHODS

### Bacterial strains and growth conditions.

*Dietzia* sp. strain DQ12-45-1b and Pseudomonas stutzeri SLG510A3-8 were isolated in our laboratory from oil fields in China and deposited in the China General Microbiological Culture Collection Center (CGMCC) (Beijing, China) under accession numbers 1.10709 and 1.15316, respectively. After the two strains were grown in GPY (10 g liter^−1^ glucose, 5 g liter^−1^ yeast extract, 10 g liter^−1^ tryptone) and LB (10 g liter^−1^ NaCl, 5 g liter^−1^ yeast extract, 10 g liter^−1^ tryptone) media, respectively, at 150 rpm and 30°C until the mid-exponential phase, they were centrifuged and washed with the minimal medium (44) three times to make inoculating seed suspensions. Afterward, the two clean seeds were separately inoculated into two flasks or mixed in one flask, each containing 100 ml minimal medium supplemented with 0.7734 g liter^−1^ C_16_. The initial cell density of each strain was an optical density at 600 nm (OD_600_) of 0.1. The culture without inoculum was taken as the control. As described in File S1 in the supplemental material, cocultivation of the two strains was also done in minimal medium supplemented with a synthetic *n*-alkane mixture consisting of C_14_, C_16_, and C_28_ or with diesel oil. Since the colonies of *Dietzia* sp. were orange and smooth, being significantly different from those of P. stutzeri, which were brown and wrinkled, samples were taken every 1 to 2 days for cell density counting by CFU until the early stationary phase. The collected data were processed using nonlinear curve fitting followed by differentiation in Origin8.5 (OriginLab Co., MA, USA) to obtain the time courses of specific growth rates.

### Analysis of C_16_ removal rates.

At the end of the cultivation period, the residual C_16_ in each flask was extracted using 20 ml *n*-hexane in a separating funnel and then was analyzed using a 5975C gas chromatograph (GC) coupled with a 7890A mass spectrometer (MS) detector (Agilent Technologies, Santa Clara, CA, USA). Helium was applied as the carrier gas at a flow rate of 25 ml min^−1^. The oven temperature was set to 100°C for 1 min and then increased to 260°C at a rate of 20°C min^−1^ and held at that temperature for 5 min. The external standard method was utilized to obtain the C_16_ concentrations in each sample. Afterward, the residual C_16_ concentration data were processed in Origin8.5 to obtain the time courses of specific C_16_ removal rates. The C_16_ removal rate was calculated as the difference in the percentage of the residual C_16_ concentration between the experimental and the control treatments in 0.7734 g liter^−1^ C_16_.

### Reconstruction and constraint-based analysis of the two-species metabolic model.

The single-species GEMs of *Dietzia* sp. DQ12-45-1b and P. stutzeri SLG510A3-8 were reconstructed as described in File S1 and then were modified by unifying the format of reactions and metabolites before being integrated to form the two-species model following the approach of Klitgord and Segrè (45). In the two-species model, the metabolites were localized into a total of five compartments as follows: (i) [d] = cytoplasm of *Dietzia* sp., (ii) [t] = extracellular space of *Dietzia* sp., (iii) [p] = cytoplasm of P. stutzeri, (iv) [s] = extracellular space of P. stutzeri, and (v) [e] = environment shared by the two strains. FBA was used to calculate the metabolic flux distribution in the reconstructed GEMs, in which the biomass reactions accounting for all known biomass constituents and their fractional contributions to the overall cellular biomass were used as the objective to calculate the flux distribution solution in the feasible flux space for the maximal growth rate. System constraints were defined with the upper bound for each metabolic reaction as 1,000 and the lower bounds as −1,000 and 0 for the reversible and irreversible reactions, respectively, except for the exchange reactions. The lower bounds of exchange reactions relating to oxygen and the medium compounds were set to −1,000, while the remaining ones were set to 0. All calculations were performed under the linear programming algorithm using the COBRA toolbox (46) on the MATLAB platform (The MathWorks Inc., Natick, MA).

### *In vitro* validation of *in silico*-predicted key interspecific compounds.

Seeds of *Dietzia* sp. DQ12-45-1b were inoculated into 100 ml minimal medium supplemented with 0.7734 g liter^−1^ C_16_ alone or with the addition of 0.0446 g liter^−1^ sodium acetate, 0.0368 g liter^−1^ sodium glutamate, 0.0283 g liter^−1^ *R*-3-hydroxybutanoic acid, 0.0318 g liter^−1^ α-ketoglutaric acid, or 0.0174 g liter^−1^ hexadecanoic acid at an initial OD_600_ of 0.1. The carbon molar contents of the five additives in the experimental treatments were equal to 2.0% of the carbon amount in 0.7734 g liter^−1^ C_16_. The pH in each treatment was adjusted to 8.0 before medium sterilization. All the cultures were incubated at 150 rpm and 30°C. Samples were taken for CFU counting and measurement of the residual C_16_ amount until the early stationary phase. To obtain the time courses of the specific growth rates and the specific C_16_ removal rates in each treatment, the collected data were processed in Origin8.5 as described above.

Seeds of P. stutzeri SLG510A3-8 were inoculated into 100 ml minimal medium supplemented with 0.872 g liter^−1^ hexadecanoic acid, 1.590 g liter^−1^ α-ketoglutaric acid, or 1.416 g liter^−1^ *R*-3-hydroxybutanoic acid at an initial OD_600_ of 0.1. The carbon molar content in each of the three compounds was equal to that in 0.7734 g liter^−1^ C_16_. The culture without any carbon source was taken as the control. All the cultures were incubated at 150 rpm and 30°C. Samples were taken for CFU counting after 24 h of cultivation.

### Label-free proteome identification and quantification.

Cells for proteomics analysis were cultivated in a 300-ml two-chamber bioreactor. As shown in Fig. S2 in File S1, the two chambers in the bioreactor were separated by a cellulose acetate filter (pore size = 0.2 μm; Sartorius Stedim Biotech GmbH, Göttingen, Germany). Seeds of *Dietzia* sp. DQ12-45-1b and P. stutzeri SLG510A3-8 were inoculated into different chambers, each of which contained 100 ml minimal medium supplemented with 0.7734 g liter^−1^ C_16_. The initial cell density for each strain was an OD_600_ of 0.1. Meanwhile, we developed the P. stutzeri monoculture control by inoculating P. stutzeri seeds into another bioreactor with an initial OD_600_ of 0.1 in each side and did the same for the *Dietzia* sp. monoculture control. All cultures were kept at 30°C with aeration. Bacterial cells were harvested at day 8 for proteome extraction. Details of proteomic sample preparation are described in File S1. The resulting tryptic peptide samples were loaded into an LC-LTQ/Orbitrap-MS (Thermo Fisher Scientific, San Jose, CA, USA). The mobile phase was comprised of solvent A (97% H_2_O, 3% acetonitrile, 1% formic acid) and solvent B (99.9% acetonitrile and 0.1% formic acid). The detection of peptide spectra was as described by Liu et al. (47). The proteome qualification was performed using the Mascot software (Matrix Science, Boston, MA, USA) against genome databases for the two strains, while the proteome quantification was based on comparison of the spectrometric spectral counts for selective proteolytic peptides (48).

### Extracellular metabolomic profiling.

Cell-free cultures of *Dietzia* sp. DQ12-45-1b, P. stutzeri SLG510A3-8, and their mixture were obtained by centrifugation of 2-ml samples at 4°C and 10,000 rpm for 10 min to remove C_16_-exposed cells. For desalinization, 10 ml methanol was added to each cell-free culture, vortexed for 5 min, and kept at 4°C for 5 h. The mixtures were then centrifuged at 20,000 rpm and 4°C for 10 min, and the supernatant was lyophilized (Freeze-Dryer; Boyikang, Beijing, China) for 4 h. The lyophilized samples were derivatized as described by Mujahid et al. (49). GC-MS analysis of the derivatized samples was performed on a GCMS-QP2010 (Shimadzu, Japan). One microliter of derivatized sample was injected into an RTx-5MS column (30 m by 0.25 mm by 0.25 μm) with helium as the carrier gas at a constant flow of 1 ml min^−1^. The inlet temperature was set at 250°C. The oven temperature was held at 80°C for 2 min, ramped to 300°C at 20°C min^−1^, and then held at 300°C for 5 min. Electron impact ionization with an ionization energy of 70 eV and transfer line temperature of 220°C was used. Mass spectra were recorded at 50 to 700 *m/z* for 5 to 19 min. Metabolites were identified from the GC-MS data based on mass spectral comparison to the standard NIST library 2.0 (National Institute of Standards and Technology, 2008) and Wiley 9 (Wiley-VCH Verlag GmbH & Co. KgaA, Weinheim, Germany), with a match threshold of >80 (with a maximum match equal to 100). Relative metabolite abundances were calculated from peak areas (unique mass) of identified metabolites from GC-MS data and then were amended using the peak area of methyl tetradecanoate to minimize instrumental errors.

### *In vitro* cultivation of the two-species consortium with the addition of predicted exchanged compounds for diesel removal efficiency test.

Seeds of *Dietzia* sp. DQ12-45-1b were inoculated into the minimal medium containing 10 g liter^−1^ diesel oil with or without the addition of P. stutzeri SLG510A3-8 and exogenous addition of 0.045 g liter^−1^ sodium acetate and 0.037 g liter^−1^ sodium glutamate. The culture without inoculum and the one inoculated with P. stutzeri SLG510A3-8 alone were taken as the two control treatments. Every treatment was performed in triplicates. All the cultures were incubated at 150 rpm and 30°C. Samples were taken weekly until the stationary phase or at the end of the culture period for *Dietzia* sp. cell density counting with CFU. At the end of the culture period, the oil removal rates were tested using the weight difference assay as described in EPA method 3500B, and diesel composition profiling was tested using GC-MS. Samples for diesel composition profiling were prepared as described previously. During the GC-MS processing, the oven temperature was set to 50°C for 1 min and then increased to 260°C at a rate of 10°C min^−1^ and held at that temperature for 5 min, after which it was sequentially increased to 280°C at 10°C min^−1^ and held at that temperature for 0.5 min.

### Statistical analysis.

All *in vitro* experiments were performed in triplicates and subjected to statistical analysis. The standard deviations were calculated in Microsoft Excel to represent the errors for the triplicate data. The *P* values between two triplicates were also calculated in Microsoft Excel, and a *P* value of <0.05 was considered statistically significant. For the proteomics analysis, proteins with a |log_2_(fold change)| of >1 were defined as high-level regulated proteins, and those with a |log_2_(fold change)| of <1 were defined as low-level regulated proteins. The significantly changed proteins were annotated according to the KEGG database to identify metabolic pathways in each strain that were influenced by the other one.

### Data availability.

The DNA sequencing data are deposited at GenBank under accession numbers CP046567.1, CP046568.1, and CP046569.1 for *Dietzia* sp. DQ12-45-1b and CP011854.1 for P. stutzeri SLG510A3-8. The mass spectrometry proteomics data reported in this paper have been deposited in the open-access database ProteomeXchange Consortium via the iProX partner repository (50) with the data set identifier PXD016754.

## Supplementary Material

Supplemental file 1

Supplemental file 2

Supplemental file 3

Supplemental file 4
