# Supplementary material for: Metabolic Exchange with Non-Alkane-Consuming Pseudomonas stutzeri SLG510A3-8 Improves n-Alkane Biodegradation by the Alkane Degrader Dietzia sp. Strain DQ12-45-1b
Source: Appl Environ Microbiol. 2020 Apr 1;86(8):e02931-19. doi: 10.1128/AEM.02931-19 (PMC7117941; doi:10.1128/AEM.02931-19)
Supplement: Supplemental file 1 [file AEM.02931-19-s0001.pdf]

Supplementary File S3 Contents

Supplementary Results 1
Metabolic reconstruction of *Dietzia* sp. DQ12-45-1b and model analysis 1
Metabolic reconstruction of *P. stutzeri* SLG510A3-8 and model analysis 3
Reconstruction and supplementary characterization of the two-species model 5
in vitro cultivation of *Dietzia* sp. DQ12-45-1b and *P. stutzeri* SLG510A3-8 on *n*-alkanes with various chain lengths 7
Supplementary Materials and Methods 9
GEM reconstruction of *Dietzia* sp. DQ12-45-1b 9
GEM reconstruction of *P. stutzeri* SLG510A3-8 9
Biomass composition determination 10
in vitro cultivating experiments to obtain the specific biomass growth rates and substrate consuming rates 11
BIOLOG experiments 11
Label-free proteomic sample preparation 12
in vitro cultivation of *Dietzia* sp. DQ12-45-1b and *P. stutzeri* SLG510A3-8 on various *n*-alkanes 13
Supplementary References 13
Supplementary Tables 16
Table S1 16
Table S2 16
Table S3 17
Table S4 17
Table S5 18
Table S6 20
Table S7 20
Table S8 21
Table S9 22
Supplementary Figures 25
Figure S1 25
Figure S2 26
Figure S3 27
Figure S4 28
Figure S5 29
Figure S6 30
Figure S7 31
Figure S8 32
Figure S9 33

Supplementary Results

Metabolic reconstruction of *Dietzia* sp. DQ12-45-1b and model analysis

The initial metabolic model of *Dietzia* sp. DQ12-45-1b was created from a list of reactions collected based on genomic alignment with three phylogenetically close species, *C. glutamicum* ATCC13032, *M. tuberculosis* H37Rv and *S. coelicolor* A3(2), whose GEMs were available (52-54). As described in Supplementary Materials and Methods, the initial model was extensively curated based on its annotated genome, various genetic and chemical databases (i.e. KEGG, SEED, CELLO, TCDB, and MetaCyc), literatures and *in situ* biochemical information (i.e. biomass compositions and depletion of glucose and C_{16} during the primary-phase growth), and an iterative gapfinding-gapfilling process. The refined model was finally validated by comparison *in silico* prediction with the physiological data and Biolog substrate utilization data. At the end, the completed GEM, namely *i*BH925, consisting of 925 genes out of 3633 protein-coding genes (25.46%), 937 metabolites and 1316 reactions distributed over two compartments (cytoplasm and extracellular) was constructed (Supplementary file S1). The gene representing percentage of *i*BH925 was higher than its reference models, which were 9.23%, 16.43% and 6.73% for *C. glutamicum* ATCC13032 model, *i*NJ661m and *S. coelicolor* A3(2) model, respectively.

*i*BH925 contained 1019 metabolic reactions, 148 transport reactions, 146 exchange reactions, one sink reaction, and two biomass equations. The intracellular metabolic reactions were categorized into 14 different metabolic subsystems, according to KEGG pathway database. As shown in Fig S5, the sum of the five largest subsystems accounted for over 80% of the total number of metabolic reactions (83.60%), of which the largest group of reactions, 25.15% of the total number, belonged to lipid metabolism and the second to fifth largest groups of reactions pertained to metabolism of cofactors and vitamins (17.09%), amino acid metabolism (15.52%), carbohydrate metabolism (13.75%), and nucleotide metabolism (12.08%), respectively.

Biomass equation is very important for a metabolic network, since it is typically considered as the objective function during FBA. In this work, the two biomass equations in *i*BH925 representing

cellular growth of *Dietzia* sp. DQ12-45-1b on glucose and C_{16}, respectively, were formed according to its biomass compositions on the two carbon sources. The composition of different cellular components was determined as described in supplementary file S1. The contents of lipids, protein, DNA, RNA, carbohydrates and ash, as well as nucleotide, amino acid and lipid compositions were determined from the samples taken at the late exponential phase of the aerobic cultivation of the strain on glucose and C_{16}, respectively, while carbohydrate and co-factor compositions were referred and modified from data in *i*NJ661 (55), which was the original data source for *i*NJ661m.

In order to validate and evaluate the reconstructed network, the predicted *in silico* growth rates and carbon source utilization obtained by FBA simulation of *i*BH925 were compared with the *in vitro* experimental growth and Biolog data in the study. *Dietzia* sp. DQ12-45-1b was experimentally grown on glucose and C_{16}, respectively. The time courses of growth curves and residual glucose/C_{16} contents are shown in Fig S1a and S1c, and the experimental data was processed using nonlinear curve fitting followed by differentiation in Origin 8.5 (OriginLab Co., MA, US) to obtain the time courses of specific growth rates (h^{-1}) and substrate consuming rate (mmol g^{-1} h^{-1}) on the two carbon sources (Fig S1b and S1d). The highest specific growth rates were determined to be 1.37×10^{-2} h^{-1} and 2.81×10^{-2} h^{-1}, respectively, with the specific glucose and C_{16} uptake rates of 0.15 mmol g^{-1} h^{-1} and 7.18×10^{-2} mmol g^{-1} h^{-1} at these time points. The *in silico* specific growth rates of *i*BH925 on minimal medium were calculated to be 1.52×10^{-2} h^{-1} and 2.38×10^{-2} h^{-1}, respectively, using FBA, with glucose and C_{16} input fluxes setting to be the same as the experimentally determined values (Table S2). The differences between the *in silico* prediction and the *in vitro* measurements were lower than 20%, indicating that *i*BH925 was effective for the simulation of cell growth. Interestingly, *i*BH925 grew 9.87% faster in glucose than *in vitro* experimental data for *Dietzia* sp., while it grew 18.07% slower in C_{16} than *in vitro* experimental data for the strain. The difference in growth rates might be explained by the inexact formulation of biomass equations or missing adaptation to the two compounds as primary carbon sources (56). A Biolog assay was performed for *Dietzia* sp. by cultivating the strain on 71 carbon sources in the GENIII microplate. As shown in Table S8, 63 out of the 71 tested substrates were aerobically utilized by *Dietzia* sp., and *i*BH925 was able to simulate growth with 41 of the 63 Biolog assayed compounds as sole carbon sources. The 22 substrates posed false negative (compounds utilized *in vitro* and failed to be simulated for growth *in silico*), of which

eight compounds (including stachyose, N-acetyl-β-D-mannosamine, L-fucose, D-arabitol, D-galacturonic acid, D-glucuronic acid, p-hydroxy phenyacetic acid and D-malic acid) were not utilized in the model because functional genes responding for their transportation and/or metabolism were not annotated in the strain, eight another compounds (including D-turanose, β-methyl-D-glucoside, D-fucose, glycyl-L-proline, glucuronamide, methylpyruate, bromo-succinic acid and Tween 40) were not available for *i*BH925 due to unknown information on their metabolic pathways, and the remaining five compounds (D-salicin, D-glucose 6-phosphate, D-fructose 6-phosphate, mucic acid and D-saccharic acid) could not be elucidated. Nevertheless, the comparison of Biolog data with *i*BH925 growth simulations indicates that the core metabolic network of *Dietzia* sp. DQ12-45-1b has been properly reconstructed.

Metabolic reconstruction of *P. stutzeri* SLG510A3-8 and model analysis

A combination of automatic and manual approaches was used in the GEM reconstruction process for *P. stutzeri* SLG510A3-8. Briefly, using the metabolic network of *P. stutzeri* A1501 (*i*PB890) as the template, a draft metabolic network of *P. stutzeri* SLG510A3-8 was reconstructed. Strain A1501 was isolated from the rice rhizosphere and could provide the plant with fixed nitrogen and phytohormones (47). The genome of strain A1501 was used to develop the first genome-scale metabolic model (*i*PB890) for *P. stutzeri*, which was the sole model for the species until now. The reactions from the template model which were either essential non-gene associated reactions or associated to genes which were found in the bidirectional pBLAST search were added to the draft model of *P. stutzeri* SLG510A3-8. The organism-specific model was reliable source of information since it had previously undergone manual refinements during the process of reconstruction and validation against different database, literature and experimental data. After the replacement, the draft model of *P. stutzeri* SLG510A3-8 was developed, and it involved 857 genes, 1103 reactions, and 800 metabolites. In the second stage, the draft network was refined through the modification of the biomass equation based on biomass composition information obtained from our experimental measurements and literatures, as well as an iterative and curative process of gap finding and gap filling via algorithms in COBRA toolbox in MATLAB software. Finally, the complete GEM of the strain, namely *i*BH983, was composed of 983 genes, 1193 reactions and 943 metabolites

(Supplementary File S2). According to previous report, *P.stutzeri* strain SLG510A3-8 has a 4.6 Mb circular chromosome containing 4379 protein-coding genes (27). *i*BH983 presented 22.45% genome information of *P.stutzeri* SLG510A3-8, and the gene presenting percentage of *i*BH983 was higher than that of *i*PB890 (21.74%), indicating that *i*BH983 was more comprehensive to reflect the phenotype of the corresponding *P. stutzeri* strain than the reference model.

Model *i*BH983 consists of 984 intracellular metabolic reactions, 111 transport reactions, 96 exchange reactions and two biomass equations. As shown in Fig S6, all the intracellular metabolic reactions were classified into 13 different subsystems, according to the KEGG Pathway Database. The sum of the five largest subsystems accounts for over 80% of the total number of metabolic reactions (80.18%), of which reactions related to amino acid metabolism and lipid metabolism occupied the largest and the second largest groups respectively (21.04% and 17.78%). The two biomass equations in *i*BH983 were formed from the major components of *P. stutzeri* SLG510A3-8 cellular dry matter grown on glucose and nonsugar at the exponential phase, including DNA, RNA, lipids, proteins, carbohydrates, co-factors and vitamins. The biomass composition of strain SLG510A3-8 on glucose was assumed to be similar to that of strain A1501 with some modification based on our experimental measurements as described in supplementary file S2. Glycogen was included in the composition, but it was reported that this component was generally produced only in the post-stationary growth phase of *Pseudomonas* strains when the carbon source was nonsugar (57). Therefore, a biomass equation for nonsugar carbon source was formed by omitting glycogen from the biomass equation for glucose in the model. Detailed information on the formation of the two *P. stutzeri* SLG510A3-8 biomass equations are shown in Supplementary file S2.

The reconstructed model was verified based on the experimental data of *P. stutzeri* SLG510A3-8 growth rate measurement on glucose and carbon source utilization on Biolog assay. In the study, *P. stutzeri* SLG510A3-8 was grown on glucose other than C_{16} for growth data collection, since the cells were unable to accumulate on MF medium with C_{16} as the sole carbon source according to our previous experiment (data not shown). In this work, cells of *P. stutzeri* SLG510A3-8 were found to be able to quickly remove 1 g L^{-1} glucose in 7 hours, and the highest specific growth rate of the strain was determined to be 0.25 h^{-1} with the specific glucose uptake rates of 2.20 mmol g^{-1} h^{-1} at this time points (Fig S7). The *in silico* specific growth rate of *i*BH983 on minimal medium were

calculated to be 0.22 h^{-1} via FBA when the flux lower bound for D-glucose exchange reaction in the format was set to be 2.20. The *in silico* predicted value was 13.39% lower than *in vitro* experimental data. The percent difference for *i*BH983 was higher than that for its reference model *i*PB890 (4.04%) which was might because the biomass equation in *i*BH983 for strain SLG510A3-8 was less exact than that in *i*PB890 for strain A1501, but the value was similar with that for *i*MO1056, the GEM of *P. aeruginosa* PAO1 (58), indicating that the reconstructed model was valuable for cell growth simulation. The other validating experiment was the Biolog assay, in which the availability of 95 carbon compounds for *P. stutzeri* SLG510A3-8 was tested through a high throughput analysis. As shown in Table S9, 47 out of the 95 carbon substrates were oxidized by strain SLG510A3-8. In detail, the strain was able to *in vitro* utilize glucose, maltose and glycogen, but was unable to grow on some other saccharides, such as arabinose, cellobiose, fructose, lactose, mannose, raffinose, rhamnose and sucrose; there were 35 acids and acid methyl esters in the platform, among which 28 were *in vitro* available to *P. stutzeri* SLG510A3-8 indicating that the strain preferred to utilize small-molecular acids; some essential amino acids were *in vitro* unavailable to strain SLG510A3-8 such as L-histidine, but its downstream product urocanate was able to be utilized by the strain. By using FBA, the 95 carbon compounds were tested on *i*BH983, and the biomass growth was successfully simulated for 39 compounds, of which 37 compounds were true positive (compounds utilized *in vitro* and *in silico*). The ten false negative carbon sources (Tween40, Tween80, pyruvic acid methyl ester, succinic acid mono-methyl ester, D-glucuronic acid, α-ketovaleric acid, sebacic acid, bromosuccinic acid, glucuronamide and uridine) gave *in silico* non-growth phenotype due to the lack of knowledge on their metabolic pathways, while the two false positive carbon sources (L-ornithine and L-threonine) were wrongly gave *in silico* growth phenotype because the correctly lacked L-ornithine and L-threonine transport and/or metabolic reactions might be incorrectly added in *i*BH983. Generally, the agreement of *i*BH983 with *in vitro* experimental data was 87.37%, indicating that *i*BH983 could effectively represent the aerobic metabolism of *P. stutzeri* SLG510A3-8 on a variety of common substrates.

Reconstruction and supplementary characterization of the two-species model

To obtain the metabolic model for the microbial consortium of *Dietzia* sp. DQ12-45-1b and *P.*

stutzeri SLG510A3-8, *i*BH925 and *i*BH983 were reconciled to unify the metabolites and reaction formats followed by the network integration as shown in Fig S8. The final model for the microbial consortium, namely *i*BH1908, had five compartments, of which compartments d and t represented cytoplasm and surround environment of *Dietzia* sp. DQ12-45-1b, respectively, compartments p and s represented cytoplasm and surround environment of *P. stutzeri* SLG510A3-8, respectively, and compartment e represented environment shared by the two strains. In the model, transportation of metabolites through compartments d/p and t/s were defined as transport reactions, through compartments t/s and e were defined as shuttle reactions, and through compartment e and environment out of the closed system were defined as exchange reactions. Despite 257 transport reactions, 237 shuttle reactions, 168 exchange reactions and four biomass equations, *i*BH1908 contained 2004 metabolic reactions categorized into 80 specific metabolic pathways based on their functional roles in the KEGG pathway classification scheme, and subsystems of lipid metabolism, amino acid metabolism, and metabolism of cofactors and vitamins were found to be the top three biggest metabolic groups (Fig S9a), which accounting for 21.52%, 18.17% and 15.93% of the total number of metabolic reactions, respectively. Reactions related to lipid metabolism were found to be the most important in the joint model, which agreed with the fact that *Dietzia* sp. DQ12-45-1b was active of utilizing a wide range of petroleum hydrocarbons for growth (24) and complete sets of genes for fatty acid biosynthesis and degradation were detected in *P. stutzeri* SLG510A3-8 genome (27). By analyzing model *i*BH1908 using singleGeneDeletion algorithm in FBA, it was found that 346 genes were essential for the synthetic microbial consortium under minimal C_{16} medium condition. The distribution of the essential genes is shown in Fig S9b. Around 65.31% genes in glycan biosynthesis and metabolism and 60.87% genes in metabolism of terpenoids and polyketides were essential for *i*BH1908 growth, indicating that the pathways of glycan, terpenoids and polyketides metabolism did not have many alternative routes and were quite rigid in the microbial consortium.

in vitro **cultivation of** ***Dietzia*** **sp. DQ12-45-1b and** ***P. stutzeri*** **SLG510A3-8 on** ***n*****-alkanes with various chain lengths**

We believed that the synthetic microbial consortium of *Dietzia* sp. DQ12-45-1b and *P. stutzeri* SLG510A3-8 did not only have the synergistic biodegradation capability on C_{16}, but also could cooperate for the greater recovery of *n*-alkanes with other chain lengths. In the study, the two bacterial strains were aerobically co-cultivated on the minimal medium suplemented with *n*-alkane mixture of C_{14}, C_{16} and C_{28}. The co-culture and the monocultures of each strains were kept at 30ºC for 30 days by taking cell-free cultures as the negative control. Cell densities in terms of CFU mL^{-1} and the residual *n*-alkane abundances were measured as described in the Materials and Methods. As shown in Fig S3a, the cell densities of *P. stutzeri* decreased to a very low level when the strain was grown on *n*-alkanes alone, around $(1.85\pm0.19)\times10^4$ CFU mL^{-1}, but its cell density gradually increased to $(2.10\pm0.10)\times10^6$ CFU mL^{-1} in the presence of strain *Dietzia* sp., which was in agreement with the previous finding in Fig 1a. It was observed that *Dietzia* sp. grew well on *n*-alkanes without the existence of *P. stutzeri*, and the *Dietzia* cell densities increased to be $(1.20\pm0.20)\times10^9$ and $(1.73\pm0.75)\times10^9$ CFU mL^{-1}, respectively, at the end of the cultivation period, but the cell growth rate was slightly higher when its was exposed to *P. stutzeri*. GC-MS analysis of the residual *n*-alkanes revealed that C_{14}, C_{16} and C_{28} were all available to *Dietzia* sp. with the removal efficiencies of 52.15±1.28%, 75.75±2.72% and 11.03±3.80%, respectively, which was in agreement with previous reports (24), while the three *n*-alkane compounds were not preferred by *P. stutzeri* with the removal efficiencies of 9.38±2.69%, 6.30±2.97%, 4.45±2.48%, respectively (Fig S3b). Unsurprisingly, the removal efficiencies of C_{14}, C_{16} and C_{28} by the microbial consortium (57.07±2.49%, 85.48±1.00%, 18.45±0.24%, respectively) were significantly higher than those by each individual strain, respectively ($p<0.05$), proving that the two strains had synergistic effect on various *n*-alkane biodegradation. The survival of strain *P. stutzeri* SLG510A3-8 and the enhanced *n*-alkane biodegradation in the co-culture system were also considered to be due to the cross-feeding interaction of *Dietzia* sp. and *P. stutzeri*, but the exchanged metabolites between the two strains should be different from the ones in the *in vitro* cultivation on C_{16} alone, and thus the regulated *Dietzia* enzymes relating to the enhanced *n*-alkane biodegradation might not only be acetyl-CoA C-

acetyltransferase.

To learn what the exchanged compounds the two strains were during their co-cultivation on the *n*-alkane mixture consisting of C_{14}, C_{16} and C_{28}, we did a second-time constraint-based steady-state analysis of *i*BH1908, in which the reaction of ‘PSA3_biomass_equation_nonsugar’ was still taken to be the objective function, but the uptaken carbon sources were modified to be C_{14}, C_{16} and C_{28} with the uptake rates being set to be 0.11 mmol g^{-1} h^{-1}, respectively (higher than those in *i*BH925 (0.10 mmol g^{-1} h^{-1}), because C_{14}, C_{16} and C_{28} removal efficiency in the co-culture was significantly higher than that in *Dietzia* sp. DQ12-45-1b monoculture according to our *in vitro* experimental data (Fig S3b). The flux of *Dietzia* sp. DQ12-45-1b biomass equation on the *n*-alkane mixture was fixed equal to the specific growth rate obtained in *i*BH925 (0.12 h^{-1}) considering the growth curves of the strain as shown in Fig S3a. By using FBA, 16 compounds were predicted to be changed between the two submodels when the *n*-alkane mixture was used as the sole carbon input, among which, as shown in Table S7, *Dietzia* sp. DQ12-45-1b provided nine metabolites for *P. stutzeri* SLG510A3-8 growth (R-3-hydroxybutanoate, α-ketoglutarate, glycerol, hexadecanoate, hexadecenoate, L-proline, octadecanoate, formate and glycolaldehyde), while *P. stutzeri* secreted another seven compounds for *Dietzia* sp. utilization (L-glutamate, acetate, glycine, 2-oxobutanoate, L-tyrosine, L-tryptophan and uracil). To investigate the importances of *in silico* predicted seven and nine exchanged compounds on the growth of *Dietzia* sp. and *P. stutzeri*, respectively, under *n*-alkane mixture, we simulated biomass growth on models *i*BH925 and *i*BH983 following the testing strategy in the main text. The results showed that acetate and glutamate were the key metabolites secreted by *P. stutzeri* in the consortium to enhance *Dietzia* sp. growth on the *n*-alkane mixture, while R-3-hydroxybutanoate, α-ketoglutarate, glycerol and hexadecanoate were key compounds secreted by *Dietzia* sp. to support *P. stutzeri* growth. The predicted result here was in agreement with the *in silico* prediction on C_{16}, suggesting that the exchanged metabolites between the two strains were typically similar if the carbon sources for the community were of a kind.

Supplementary Materials and Methods

GEM reconstruction of *Dietzia* sp. DQ12-45-1b

To obtain the draft model of *Dietzia* sp. DQ12-45-1b, genome functions with metabolic reactions in the *Dietzia* sp. metabolic network were collected from the available predictive GEMs of three gram-positive species phylogenetically closest to genus *Dietzia*, which were *Corynebacterium glutamicum* ATCC13032 model (52), *i*NJ661m of *Mycobacterium tuberculosis* strain H37Rv (53) and *Streptomyces coelicolor* A3(2) model (54), according to homologous alignment. Also, information from the public databases were utilized for the reconstruction, such as the SEED (http://pubseed.theseed.org/) for genome auto-annotation, the KEGG (http://www/kegg.jp) for the information on known metabolic reactions, the TCDB (http://www.tcdb.org) for the search of transport reactions, the CELLO (http://cello.life.nctu.edu.tw) for subcellular localization prediction of each metabolic reactions, the MetaCyc (http://metacyc.org) and the BioPath.Explore (http://www.molecular–networks.com/biopath3) for reacting direction determination, and so on. At the same time, experiments on *Dietzia* sp. cells harvested from the two-week aerobic cultivation with glucose and C_{16} as the sole carbon sources, respectively, were operated as described in the following statements to obtain the intracellular pH values and biomass composition in the dry cells (including proteins, lipids, carbohydrates, DNA, RNA and ash) in order to develop the two functions of 'biomass synthesis' in the model. For model refinement, the metabolic reconstruction was converted into a mathematical format by using MATLAB software, and gap finding in the network were operated by using the BiomassPrecursorCheck and GapAnalysis functions in constraint-based reconstruction and analysis (COBRA) toolbox on the MATLAB platform. The gaps in the network were then subjected to the gap-filling process to allow biomass formation, in which KEGG maps and RAST model corresponding to *Dietzia* sp. DQ12-45-1b genome were used to analyze the dead-end metabolites and supply information to one-by-one gap filling.

GEM reconstruction of *P. stutzeri* SLG510A3-8

As the first stage of the model reconstruction, the draft metabolic network was reconstructed using

*i*PB890, a published GEM for *P. stutzeri* A1501 as the template (55). The reactions from the template model which were either essential non-gene associated reactions or associated to genes which are found in the bidirectional pBLAST search were added to the draft model of *P. stutzeri* SLG510A3-8. The organism-specific model was reliable source of information since it had previously undergone manual refinements during the process of reconstruction and validation against different database, literature and experimental data. The biomass equation was developed from the biomass composition information of *P. stutzeri* obtained from *in vitro* cultivation on glucose and referred from literatures. Refinement of the draft model was done in an iterative processing of gap finding and gap filling as described above.

Biomass composition determination

When the cultures arrived at the late exponential phases, the ~120 mL culture had a 2-time centrifugation-and-washing operation before being frozen at -80°C for 2 days and being dried under vacuum for 3 days. Around 0.01 g of the dry biomass was processed with TRIzol® reagent to extract RNA and the cellular proteins step by step. And the extracted RNA was purified via DNase and re-extracted via TRIzol® reagent to exclude the effect of DNA on RNA content measurement. Another ~0.01g dry biomass was processed with Tris-phenol and chloroform-isopentanol to extract DNA, and the DNA purification was operated by using the Bioteke Corporation kit. Around 0.1g of the dry biomass was used for nitrogen content measurement (N×6.25=protein) via Elementar Vario MICRO CUBE (Elementar, Langenselbold, Germany). The compositions of amino acids were referred from Jamshidi and Palsson (59) for *Dietiza* sp. DQ12-45-1b and van Duuren JB et al. (60) for *P. stutzeri* SLG510A3-8. Another 0.1 g dry biomass was used for the polar lipid extraction based on the method modified from the Folch procedure (61). The solution of the extracted polar lipids was separated into two parts: one was used for the compound profile determination via Q-Exactive™ UPLC-MS (Therom Scientific, MA, US), and the other one was blow-dried with nitrogen for the determination of the cellular lipid content (g g^{-1}). Another ~0.1g dry biomass was used for the fatty acid extraction according to our tranesterification method. The profile and the relative contents of the extracted fatty acids were determined via HPLC (SHIMADZU, Japan). To measure the ash content (g g^{-1}), ~0.1 g dry biomass was burned in a muffle furnace at 505°C for 30

min. RNA and DNA contents in the extracted solutions (ug ml^{-1}) were measured by using ultraviolet-visible spectroscopy. The formulas are listed as follows:

DNA content (ug/ml) = OD_{260} × dilution rate × 50

RNA content (ug/ml) = OD_{260} × dilution rate × 40

in vitro cultivating experiments to obtain the specific biomass growth rates and substrate consuming rates

For *Dietzia* sp. DQ12-45-1b, cells were inoculated into the minimal medium with 8 g L^{-1} glucose and 0.7734 g L^{-1} C_{16}, respectively, as the sole carbon compound (initial OD_{600}=0.1) and the cultures were aerobically kept at 150 rpm and 30 °C for 15 and 18 days, respectively. For cultures containing glucose, samples were taken every day for the measurement of cell density in terms of g L^{-1} and the responding residual glucose contents. For cultures containing C_{16}, samples were taken at Day0, Day0.5, Day2, Day6.5, Day8.5, Day11.5, Day14.5 and Day18 for the measurement of cell density (g L^{-1}) and C_{16} content. The collected data were processed using nonlinear curve fitting followed by differentiation in Origin 8.5 (OriginLab Co., MA, US) to obtain the time courses of specific growth rates (h^{-1}) and substrate consuming rate (mmol g^{-1} h^{-1}).

For *P. stutzeri* SLG510A3-8, cells were inoculated into the minimal medium with 1 g L^{-1} glucose, and aerobically cultivated for 9 hours. The cultivation condition and data analysis methods were the same with those for *Dietzia* sp. DQ12-45-1b.

BIOLOG experiments

Biolog GEN III MicroPlate™ was used for the determination of the available carbon lists of *Dietiza* sp. DQ12-45-1b. According to its introductions for use, 71 carbon source utilization assays were involved in the test panel. The cells were harvested from the LB broth at the early exponential phase (OD_{600}=6), and then were washed twice with 0.8% NaCl solution and resuspended with NaCl solution for a 30-min starvation, followed by a third washing and resuspending. The cell suspension was inoculated into the GEN III Microplate, 100ul per well with the initial OD_{595} of 0.03, and the panel was then kept at 30°C for 48 hours. The OD595 values were read at the 0th, 24th and 48th hours

for the quantification of the increased purple color, which was a reflection of the carbon source utilizing ability of the cells.

Biolog GN2 MicroPlate™ was used for the determination of the available carbon sources of *P. stutzeri* SLG510A3-8 cells. The cells were harvested from the LB medium at the early exponential phase (OD_{600}=2.2), and then were washed using 0.8% NaCl solution for 3 times. The washed cells were resuspended into the GN/GP-IF inoculating fluid (20mL per glass tube), whose final OD_{595} was adjusted to be 0.3. The cell suspension was kept at 30°C for 50-60 mins to starve the cells before the testing experiment. The testing processed was operated in a 96-well Biolog GN2 microplate test panel providing a standardized method using 95 biochemical tests. The cell suspension was inoculated into the GN2 microplate, 150 ul per well. All of the wells started out colorless when inoculated. After incubation at 30ºC for 6-24 hours, there was a burst of respiration in the wells that contained chemicals that could be oxidized and the cells reduce the tetrazolium dye forming a purple color, with the negative wells remaining colorless.

Label-free proteomic sample preparation

Briefly, the cells of *Dietzia* sp. DQ12-45-1b and *P. stutzeri* SLG510A3-8 grown in the two-chamber reactors were harvested by centrifugation at 8000 rpm for 10 min at 4ºC and washed with phosphate buffer saline (PBS) (137 mM NaCl, 2.7 mM KCl, 10 mM Na_2HPO_4, 1.8 mM KH_2PO_4, pH7.4) for three times. The bacterial pellets were resuspended in the lysis buffer (150 mM NaCl, 1 mM EDTA, 2 mM DTT, 1 mM PMSF, 10% glycerol (v:v), 1 tablet protease inhibitor cocktail per 50 mL buffer, 50 mM Tris-HCl, pH8.0) in a 2 mL EP tube containing 0.4 g glass beads. In order to break the cell wall, the tube was shaked at 2500 rpm for 30s followed by stopping on ice for 3 min, and the process was repeated for 20 times. After that, the glass beads and unbroken cells were removed by centrifugation at 3000×g for 10 min at 4ºC. The supernatants containing the cytoplasmic and membrane proteins were mixed with 5× SDS loading buffer (50% glycerol (v:v), 350 mM SDS, 506 mM DTT, 7.5 mM bromophenol blue, 250 mM Tris-HCl, pH6.8) and then be boiled for 5 min to inactivate proteins in the extracted samples. The pretreated protein solutions were kept at 4ºC for further operation. The concentration of the extracted protein in each sample was measured by Bradford protein assay with some modification (62). Then, the protein samples containing similar

protein amounts were loaded into SDS-PAGE with the stacking gel (acrylamide 5%) and separating gel (acrylamide 10%) whose thickness was 0.75 mm for protein separation, and then the gel was stained with Coomassie Brilliant Blue R-250. The proteins in each lane of the gel were split into six bins for protein digestion to obtain the tryptic peptide samples for further proteomics detection.

***in vitro* cultivation of *Dietzia* sp. DQ12-45-1b and *P. stutzeri* SLG510A3-8 on various *n*-alkanes**

The minimal medium with the addition of 6.30 g L^{-1} artificial *n*-alkane mixture was used to test the degradation capability of the synthetic microbial consortium of *Dietzia* sp. DQ12-45-1b and *P. stutzeri* SLG510A3-8 on *n*-alkanes with various chain lengths in the study. The artificial *n*-alkane mixture was composed of three medium- and long-chain *n*-alkanes with the mass proportions being similar with those in the crude oil of Daqing Oilfield, China (data not shown): 26.98% tetradecane (C_{14}), 40.16% hexadecane (C_{16}), and 32.86% octacosane (C_{28}). The seeds of each strain were washed with distilled water for three times, and then be inoculated into flasks each containing 100mL minimal medium supplemented with the *n*-alkane mixture. The initial cell density of each strain was OD_{600}=0.1. The culture without inoculum was taken as the control treatment. The experimental treatments were performed in six replicates (triplicates for sampling and data collection, and the rest three for making up), and the control treatment was performed in triplicates. Samples were taken every 10 days for cell density counting until the end of the 30th day. Similar with above description about detecting process of the residual C_{16} amounts, the residual *n*-alkane mixture in the 100 mL cultures in each treatment were extracted with *n*-hexane at the end of the cultivating period and quantified using GC-MS. Slightly different from the temperature programming above, the oven temperature was set to 100ºC for 1 min, and then it increased to 260ºC at a rate of 20 ºC min^{-1} and held at the temperature for 5 min, after which it sequentially increased to 280 ºC at a rate of 20 ºC min^{-1} and held at the temperature for another 5 min.

Supplementary References

24. Wang X, Chi C, Nie Y, Tang Y, Tan Y, Wu G, Wu X. 2011. Degradation of petroleum

hydrocarbons (C6-C40) and crude oil by a novel *Dietzia* strain. *Bioresour Technol* 102: 7755-7761.

27. Hu B, Nie Y, Geng S, Wu X. 2015. Complete genome sequence of the petroleum-emulsifying bacterium *Pseudomonas stutzeri* SLG510A3-8. *J Biotechnol* 211:1-2.

52. Shinfuku Y, Sorpitoporn N, Sono M, Furusawa C, Hirasawa T, Shimizu H. 2009. Development and experimental verification of a genome-scale metabolic model for *Corynebacterium glutamicum*. *Microbial Cell Factories* 8: 43.

53. Fang X, Wallqvist A, Reifman J. 2016. Development and analysis of an *in vivo*-compatible metabolic network of *Mycobacterium tuberculosis*. *BMC Systems Biology* 4:160.

54. Alam M, Merlo M, the STREAM Consortium, Hodgson DA, Wellington EMH, Takano E, Breitling R. 2010. Metabolic modeling and analysis of the metabolic switch in *Streptomyces coelicolor*. *BMC Genomics* 11: 202.

55. Babaei P, Marashi S, Asad S. 2015. Genome-scale reconstruction of the metabolic network in *Pseudomonas stutzeri* A1501. *Mol Biosyst* 11(11): 3022-3032.

56. Feist AM, Henry CS, Reed JL, Krummenacker M, Joyce AR, Karp PD, Broadbelt LJ, Hatzimanikatis V, Palsson BØ. 2007. A genome-scale metabolic reconstruction for *Escherichia coli* K-12 MG1655 that accounts for 1260 ORFs and thermodynamic information. *Mol Syst Biol* 3: 121.

57. Maalej H, Hmidet N, Boisset C, Buon L, Heyraud A, Nasri M. 2015. Optimization of exopolysaccharide production from *Pseudomonas stutzeri* AS22 and examination of its metal-binding ability. *J Appl Microbiol* 118(2): 356-367.

58. Oberhardt MA, Puchalka J, Fryer KE, Martins dos Santos VA, Papin JA. 2008. Genome-scale metabolic network analysis of the opportunistic pathogen *Pseudomonas aeruginosa* PAO1. *J Bacteriol* 190(8): 2790-2803.

59. Jamshidi N, Palsson BØ. 2007. Investigating the metabolic capabilities of *Mycobacterium tuberculosis* H37Rv using the in silico strain *i*NJ661 and proposing alternative drug targets. *BMC Syst Biol* 1: 26.

60. van Duuren JB, Puchalka J, Mars AE, Bucker R, Eggink G, Wittmann C, Dos Santos VA. 2013. Reconciling *in vivo* and *in silico* key biological parameters of *Pseudomonas putida* KT2440

during growth on glucose under carbon-limited condition. *BMC Biotechnol* 13: 93.

61. Folch J, Lees M, Sloane-Stanley GH. 1957. A simple method for isolation and purification of total lipids from animal tissues. *J Biol Chem* 226: 497-509.

62. Bradford MM. 1976. Rapid and sensitive method for the quantitation of microgram quantities of protein utilizing the principle of protein-dye binding. *Anal Biochem* 72: 248-254.

Supplementary Tables

Table S1. List of metabolites shared by *Dietzia* sp. DQ12-45-1b and *P. stutzeri* SLG510A3-8 in *i*BH1908

**Category**	**Metabolite**
Amino acids	D-alanine, L-alanine, L-arginine, L-asparagine, L-aspartate, L-cysteine, L-glutamine, L-glutamate, glycine, glycine betaine, L-isoleucine, L-leucine, L-lysine, L-methionine, L-ornithine, L-proline, L-threonine, L-tryptophan, L-tyrosine, L-valine
Inorganic compounds	CO_2, Co^{2+}, Cu^{2+}, Fe^{2+}, Fe^{3+}, H_2O, K^+, molybdate, Na^+, NH_4^+, NO_2, NO_3, O_2, PO_4^{3-}, SO_4^{2-}, thiosulfate, Zn^+, H^+
Small-molecular organic acids	2-hydroxybutanoic acid, 2-oxobutanoate, 4-aminobutyrate, acetate, alpha-ketoglutarate, R-3-hydroxybutanoate, citrate, formate, gluconate, D-glucarate, lactate, malate, malonate, succinate, propanoate
Fatty acids	arachidic acid, dodecanoate, hexadecanoate, hexadecenoate, octadecanoate, tetradecanoate
Saccharides	D-fructose, D-glucose, maltose
Nucleic acid	uracil
Others	choline, glycolaldehyde, glycerol, D-mannitol, urea

Table S2. Comparison of the specific growth rates of *Dietzia* sp. DQ12-45-1b obtained from *in vivo* cultivation study on glucose and C_{16} in minimal medium and *in silico* predictions for *i*BH925, respectively

	in vivo (h^{-1})	*in silico* (h^{-1})	Percent deviation
Glucose	1.37×10^{-2}	1.52×10^{-2}	9.87%
C_{16}	2.81×10^{-2}	2.38×10^{-2}	-18.07%

Table S3. List of *P. stutzeri* SLG510A3-8 proteins, relating to C_{16}, hexadecanoic acid, 3-hydroxybutanoic acid, α-ketoglutaric acid, acetate and L-glutamate metabolic pathways, whose expression were significantly high-level and low-level up- (P-HU# and P-LU#, respectively) and down-regulated (P-HD# and P-LD#, respectively) when the cells were co-cultivated with *Dietzia* sp. DQ12-45-1b on C_{16}

No.	*P. stutzeri* gene	Protein function	Average fold change (co-/mono-)
P-HU1	A3-orf04541	long-chain acyl-CoA synthetase	5.14
P-HU2	A3-orf00180	3-hydroxyacyl-CoA dehydrogenase	2.14
P-LU1	A3-orf02972	isocitrate dehydrogenase	1.83
P-LU2	A3-orf00307	flagellin	1.44
P-LD1	A3-orf03625	fumarate reductase, membrane anchor subunit	0.84
P-LD2	A3-orf03627	fumarate reductase, cytochrome b subunit	0.54
P-LD3	A3-orf00176	succinate-semialdehyde dehydrogenase	0.53
P-HD1	A3-orf01032	GABA transferase	0.43
P-HD2	A3-orf03560	acetyl-CoA C-acetyltransferase	0.39
P-HD3	A3-orf00623	glutamine synthetase	0.37
P-HD4	A3-orf03851	acetyl-CoA synthetase	0.37
P-HD5	A3-orf03628	citrate synthase	0.29
P-HD6	A3-orf03939	enyl-CoA hydratase	0.09
P-HD7	A3-orf04270	succinyl-CoA synthetase	0.09

Table S4. List of *Dietzia* sp. DQ12-45-1b proteins, relating to C_{16}, hexadecanoic acid, 3-hydroxybutanoic acid, α-ketoglutaric acid, acetate and L-glutamate metabolic pathways, whose expression were high-level and low-level up- (D-HU # and D-LU#, respectively) and down-regulated D-HD# and D-LD#) when the cells were co-cultivated with *P. stutzeri* SLG510A3-8 on C_{16}

No.	*Dietzia* sp. gene	Protein function	Average fold change (co-/mono-)
D-HU1	Contig46-orf02865	malate dehydrogenase	3.40
D-HU2	Contig46-orf02032	long-chain acyl-CoA synthetase	3.38
D-LU1	Contig46-orf00979	GABA transferase	1.97
D-LU2	Contig46-orf00397	glutamate synthase	1.89
D-LU3	Contig46-orf01974	glutamine synthetase	1.85
D-LU4	Contig46-orf01422	fumarate reductase	1.08
D-LD1	Contig46-orf03652	succinyl-CoA synthetase	0.72
D-HD1	Contig46-orf00375	aldehyde dehydrogenase	0.38
D-HD2	Contig46-orf04261	enyl-CoA hydratase	0.18

Table S5. List of extracellular metabolites and their chromatographic peak area fractions (%) in the cell-free solution of the co-culture system and pure cultures of *Dietzia* sp. DQ12-45-1b and *P. stutzeri* SLG510A3-8, respectively, when they were exposed to C_{16}

Compounds	Co-culture	*Dietzia* sp. DQ12-45-1b	*P. stutzeri* SLG510A3-8
Unknown1	0.30±0.11	0.82±0.28	0.28±0.08
Acetamide	0.18±0.07	0.38±0.13	0.16±0.04
D-lactic acid	0.83±0.43	0.71±0.48	0.78±0.25
Glycolic acid	0.04±0.01	0.05±0.01	0.06±0.05
Unknown2	ND	ND	0.01±0.02
Unknown3	0.50±0.23	1.36±0.52	0.49±0.17
Unknown4	0.10±0.04	0.09±0.03	0.10±0.03
Unknown5	0.08±0.03	0.07±0.03	0.08±0.02
Unknown6	0.11±0.04	0.01±0.01	0.09±0.02
Unknown7 *	0.11±0.04	0.13±0.08	0.05±0.03
Oxalate	0.19±0.05	0.24±0.09	0.32±0.15
3-hydroxybutyric acid ǂ	0.02±0.01	ND	0.02±0.01

Dodecamethylpentasiloxane	5.28±2.94	2.98±0.93	3.83±1.72
Glutamate	0.22±0.09	0.39±0.13	0.30±0.07
Ethanolamine	1.51±0.60	3.68±1.31	1.36±0.40
Unknown8	1.59±1.00	1.52±1.13	0.85±0.24
Unknown9	1.27±1.08	0.79±0.40	0.50±0.20
Unknown10 ǂ	3.46±1.36	0.11±0.03	2.85±0.47
2-ketoglutaric acid	0.01±0.01	0.36±0.15	ND
Tetradecanoate	5.17±1.84	4.73±1.66	4.80±1.40
Unknown11	0.36±0.15	0.50±0.18	0.37±0.20
Unknown12	6.74±2.42	4.97±2.35	6.34±2.31
Unknown13	0.37±0.23	0.27±0.16	0.33±0.11
Hexadecanoate	0.71±0.49	0.56±0.53	0.36±0.14
Inositol	0.03±0.02	0.10±0.04	0.01±0.00
Hexadecanamide	0.05±0.02	0.03±0.01	0.05±0.02
Octadecanoic acid	0.61±0.56	0.59±0.38	0.31±0.10
Unknown14	0.42±0.27	0.25±0.08	0.28±0.11
Unknown15	0.68±0.51	0.56±0.24	0.57±0.26
9-Octadecenamide	1.77±0.50	1.24±0.58	2.02±0.74
Octadecanamide	0.07±0.02	0.04±0.02	0.07±0.03
Unknown16	0.38±0.23	0.37±0.27	0.23±0.04
Unknown17	0.46±0.29	0.31±0.13	0.16±0.01
1,2-benzenedicarboxylic acid	0.06±0.04	0.05±0.04	0.03±0.02
Unknown18	0.12±0.07	0.06±0.03	0.03±0.01
Unknown19	0.50±0.32	0.21±0.11	0.10±0.02
Unknown20	0.28±0.03	0.30±0.12	0.42±0.24
Trehalose/Sucrose	0.70±0.54	0.06±0.05	0.04±0.03
Unknown21	0.01±0.01	0.03±0.05	0.07±0.05
Unknown22	0.05±0.06	0.06±0.05	0.07±0.06
Unknown23	0.07±0.09	0.27±0.25	0.49±0.44

Unknown24	0.25±0.21	0.07±0.10	0.16±0.20
Phosphate	64.36±14.92	70.66±11.62	70.54±8.96

* Significantly different between that in the co-culture system and *P. stutzeri* SLG510A3-8 pure culture (p<0.05)

ǂ Significantly different between that in the co-culture system and *Dietzia* sp. DQ12-45-1b pure culture (p<0.05)

Table S6. Relative abundances of *Dietzia* alkane hydroxylases in *Dietzia* sp. DQ12-45-1b monoculture and the co-culture systems on C_{16}

	**Relative abundance (%)**		
**Protein name**	**Co-culture**	***Dietzia* sp. monoculture**	***p*-value**
AlkW1	0.07±0.02	0.08±0.08	0.81
AlkW2	0.20±0.05	0.11±0.10	0.24
CYP153	0.67±0.15	0.16±0.13	0.06

Table S7. The interspecific metabolites that induced the growth of *P. stutzeri* SLG510A3-8 and *Dietzia* sp. DQ12-45-1b on the *n*-alkane mixture using *i*BH1908.The compounds in bold represent the key compounds for the two strains.

**Reaction description**	**Formula**	**Flux (mmol g^{-1} h^{-1})**	**Reaction description**	**Formula**	**Flux (mmol g^{-1} h^{-1})**
**(R)-3-Hydroxybutanoate[t]** shuttle	bhb[t] <=> bhb[e]	1.63	**L-glutamate[s]** shuttle	glu-L[s] <=> glu-L[e]	1.83
**2-Oxoglutarate[t]** shuttle	akg[t] <=> akg[e]	0.97	**Acetate[s]** shuttle	ac[s] <=> ac[e]	1.65
**Glycerol[t]** shuttle	glyc[t] <=> glyc[e]	0.38	Glycine[s] shuttle	gly[s] <=> gly[e]	5.79×10^{-2}
**Hexadecanoate[t]** shuttle	hdca[t] <=> hdca[e]	9.59×10^{-2}	2-oxobutanoate[s] shuttle	2obut[s] <=> 2obut[e]	2.18×10^{-2}
Hexadecenoate[t] shuttle	hdcea[t] <=> hdcea[e]	3.53×10^{-2}	L-Tyrosine[s] shuttle	tyr-L[s] <=> tyr-L[e]	7.90×10^{-3}
L-Proline[t] shuttle	pro-L[t] <=> pro-L[e]	1.51×10^{-2}	L-Tryptophan[s] shuttle	trp-L[s] <=> trp-L[e]	4.99×10^{-3}
Octadecanoate[t] shuttle	ocdca[t] <=> ocdca[e]	1.90×10^{-4}	Uracil[s] shuttle	ura[s] <=> ura[e]	5.79×10^{-4}

Formate[t] shuttle	for[t] <=> for[e]	3.87×10^{-5}
Glycolaldehyde[t] shuttle	gcald[t] <=> gcald[e]	1.69×10^{-5}

Table S8. The results of a Biolog study of *Dietzia* sp. DQ12-45-1b were compared with *in silico* viability predictions for *i*BH925 obtained via FBA.

**Label**	**Carbon source**	***in vivo***	***in silico***	**Label**	**Carbon source**	***in vivo***	***in silico***
A1	Water	-	×	E1	Gelatin	-	×
A2	Dextrin	+++	√	E2	Glycyl-L-Proline	+++	×
A3	D-Maltose	+++	√	E3	L-Alanine	+++	√
A4	D-Trehalose	++	√	E4	L-Arginine	+++	√
A5	D-cellobiose	-	×	E5	L-Aspartic acid	+++	√
A6	Gentiobiose	-	×	E6	L-Glutamic acid	+++	√
A7	Sucrose	+	√	E7	L-histidine	+++	√
A8	D-Turanose	+	×	E8	L-Pyroglutamic acid	+++	√
A9	Stachyose	+	×	E9	L-serine	+++	√
B1	D-Raffinose	-	×	F1	Pectin	-	
B2	α-D-Lactose	+	√	F2	D-Galacturonic acid	+++	×
B3	D-Melibiose	+	√	F3	L-Galactonic acid lactone	+++	√
B4	β-methyl-D-glucoside	+	×	F4	D-Gluconic acid	+++	√
B5	D-salicin	+	×	F5	D-Glucuronic acid	+++	×
B6	N-Acetyl-D-Glucosamine	+++	√	F6	Glucuronamide	+++	×
B7	N-Acetyl-β-D-Mannosamine	+	×	F7	Mucic acid	+++	×
B8	N-Acetyl-D-Galactosamine	+	√	F8	Quinic acid	+++	√
B9	N-Acetyl Neuraminic acid	-	×	F9	D-Saccharic acid	+++	×
C1	α-D-Glucose	+++	√	G1	p-Hydroxy-phenylacetic acid	+++	×
C2	D-Mannose	+	√	G2	Methyl pyruvate	+++	×
C3	D-Fructose	+++	√	G3	D-lactic acid methyl ester	-	×
C4	D-Galactose	++	√	G4	L-lactic acid	+++	√
C5	3-Methyl Glucose	+	×	G5	Citric acid	+++	√
C6	D-Fucose	++	×	G6	α-keto-glutaric acid	+++	√
C7	L-Fucose	++	×	G7	D-Malic acid	+++	×
C8	L-Rhamnose	+	√	G8	L-Malic acid	+++	√
C9	Inosine	-	×	G9	Bromo-succinic acid	++	×
D1	D-Sorbitol	+	√	H1	Tween 40	+++	×
D2	D-Mannitol	+++	√	H2	γ-Amino-butyric acid	+++	√

D3	D-Arabitol	+++	×	H3	α-Hydroxy-butyric acid	+++	√
D4	myo-Inositol	+	√	H4	β-Hydroxy-D,L-Butyric acid	+++	√
D5	Glycerol	+++	√	H5	α-Keto-butyric acid	++	√
D6	D-Glucose-6-PO_4	+	×	H6	Acetoacetic acid	++	√
D7	D-Fructose-6-PO_4	++	×	H7	Propionic acid	+++	√
D8	D-Aspartic acid	+++	√	H8	Acetic acid	+++	√
D9	D-Serine	+	√	H9	Formic acid	+++	√

Table S9. The results of a Biolog study of *Pseudomonas stutzeri* SLG510A3-8 were compared with *in silico* viability predictions for *i*BH983 obtained via FBA

Label	Compound	*in vitro*	*in silico*	Label	Compound	*in vitro*	*in silico*
A1	Water	-	×	E1	p-Hydroxyphenylacetic Acid	-	×
A2	α-Cyclodextrin	-	×	E2	Itaconic Acid	+++	√
A3	Dextrin	++	√	E3	α-Ketobutyric Acid	++	√
A4	Glycogen	++	√	E4	α-ketoglutaric Acid	+++	√
A5	Tween 40	++	×	E5	α-ketovaleric Acid	+	×
A6	Tween 80	++	×	E6	D,L-Lactic Acid	++	√
A7	N-Acetyl-D-Galactosamine	-	×	E7	Malonic Acid	++	√
A8	N-Acetyl-D-Glucosamine	-	×	E8	Propionic Acid	+++	√
A9	Adonitol	-	×	E9	Quinic Acid	-	×
A10	L-Arabinose	-	×	E10	D-Saccharic Acid	+++	√
A11	D-Arabitol	-	×	E11	Sebacic Acid	+	×
A12	D-Cellobiose	-	×	E12	Succinic Acid	++	√
B1	i-Erythritol	-	×	F1	Bromosuccinic Acid	++	×
B2	D-Fructose	+	√	F2	Succinamic Acid	-	×
B3	L-Fucose	-	×	F3	Glucuronamide	++	×
B4	D-Galactose	-	×	F4	L-Alaninamide	++	√
B5	Gentiobiose	-	×	F5	D-Alanine	++	√
B6	α-D-Glucose	++	√	F6	L-Alanine	++	√
B7	m-Inositol	-	×	F7	L-Alanyl-Glycine	-	×

B8	α-D-Lactose	-	×	F8	L-Asparagine	+++	√
B9	Lactulose	-	×	F9	L-Aspartic Acid	+	√
B10	Maltose	++	√	F10	L-Glutamic Acid	+++	√
B11	D-Mannitol	++	√	F11	Glucyl-L-Aspartic Acid	-	×
B12	D-Mannose	-	×	F12	Glycyl-L-Glutamic Acid	-	×
C1	D-Melibiose	-	×	G1	L-Histidine	-	×
C2	β-Methyl-D-Glucoside	-	×	G2	Hydroxy-L-Proline	-	×
C3	D-Psicose	+	√	G3	L-Leucine	+++	√
C4	D-Raffinose	-	×	G4	L-Ornithine	-	√
C5	L-Rhamnose	-	×	G5	L-Phenylalanine	-	×
C6	D-Sorbitol	-	×	G6	L-Proline	++	√
C7	Sucrose	-	×	G7	L-Pyroglutamic Acid	++	√
C8	D-Trehalose	-	×	G8	D-Serine	-	×
C9	Turanose	-	×	G9	L-Serine	-	×
C10	Xylitol	-	×	G10	L-Threonine	-	√
C11	Pyruvic Acid Methyl Ester	++	×	G11	D,L-Carnitine	-	×
C12	Succinic Acid Mono-Methyl Ester	+	×	G12	γ-Aminobutyric Acid	+	√
D1	Acetic Acid	++	√	H1	Urocanic Acid	+	√
D2	Cis-Aconitic Acid	++	√	H2	Inosine	+	√
D3	Citric Acid	++	√	H3	Uridine	+	×
D4	Formic Acid	+	√	H4	Thymidine	-	×
D5	D-Galactonic Acid Lactone	-	×	H5	Phenylethylamine	-	×
D6	D-Galacturonic Acid	++	√	H6	Putrescine	++	√
D7	D-Gluconic Acid	++	√	H7	2-Aminoethanol	-	×
D8	D-Glucosaminic Acid	-	×	H8	2,3-Butanediol	-	×
D9	D-Glucuronic Acid	++	×	H9	Glycerol	+++	√
D10	α-Hydroxybutyric Acid	+	√	H10	D,L,α-Glycerol Phosphate	-	×
D11	β-Hydroxybutyric Acid	++	√	H11	α-D-Glucose-1-Phosphate	-	×

D12	γ-Hydroxybutyric Acid	-	×	H12	D-Glucose-6-Phosphate	-	×

Supplementary Figures

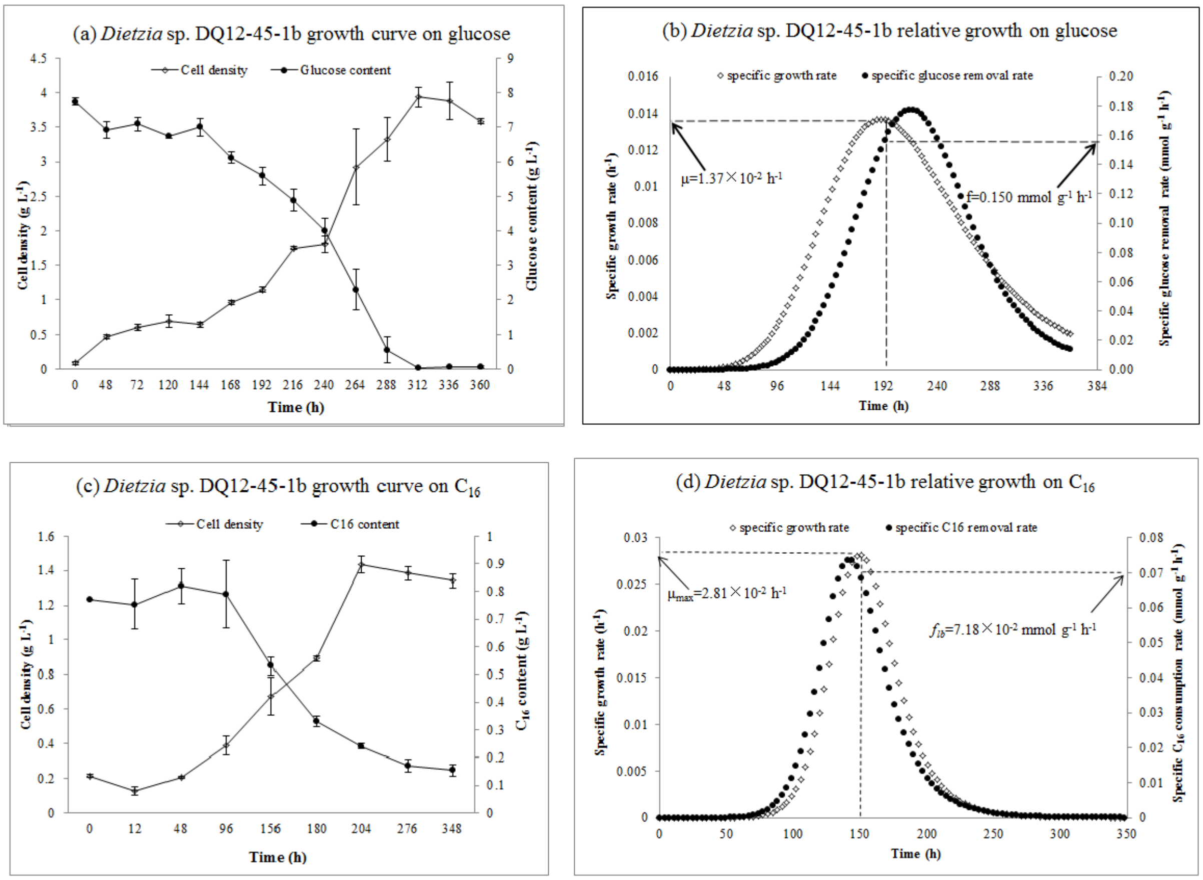

Figure S1. Growth of *Dietzia* sp. DQ12-45-1b cells with glucose and C_{16} as the sole carbon sources, respectively. The time courses of cell density (g L^{-1}) grown on glucose and residual glucose content (g L^{-1}) (a); the time courses of the relative cell growth rate (h^{-1}) and glucose relative consumption rate (mmol g^{-1} h^{-1}) for the strain (b); the time courses of cell density (g L^{-1}) grown on C_{16} and residual C_{16} content (g L^{-1}) (c); The time courses of the relative cell growth rate (h^{-1}) and C_{16} relative consumption rate (mmol g^{-1} h^{-1}) for the strain (d).

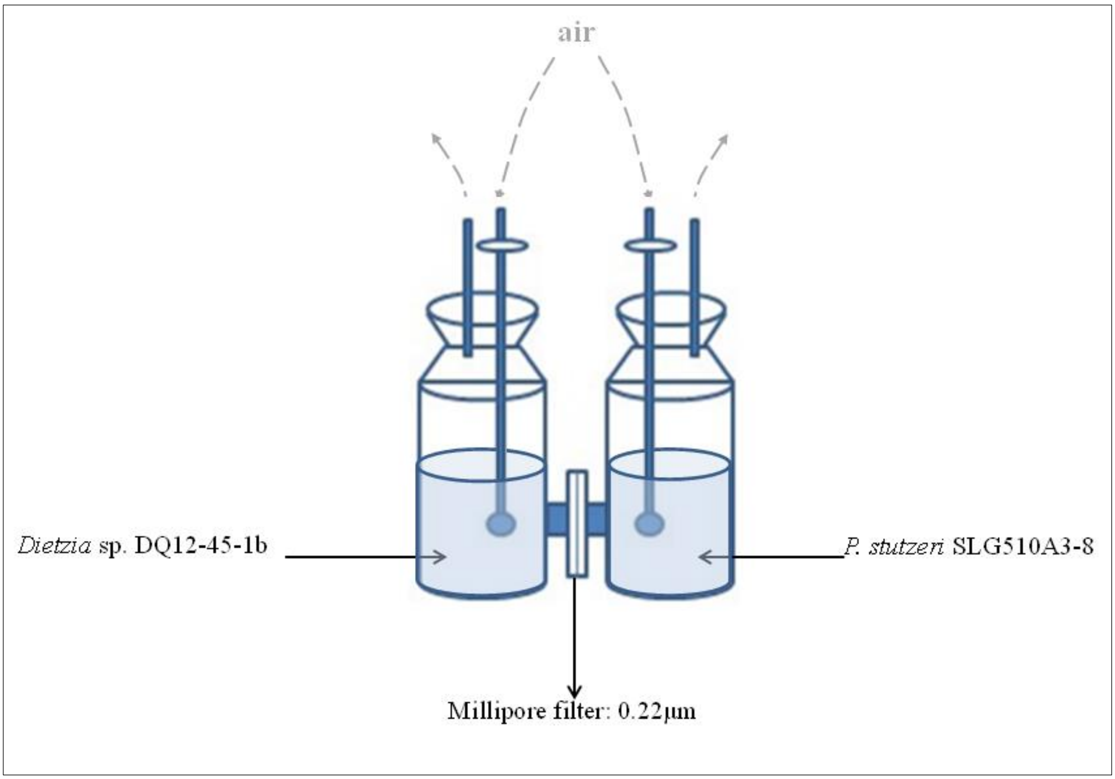

Figure S2. Diagram of the H-shape two-chamber reactor in which strains *Dietzia* sp. DQ12-45-1b and *P. stutzeri* SLG510A3-8 were separated in two sub-cells by a cellulose acetate filter with the pore size of 0.2μm.

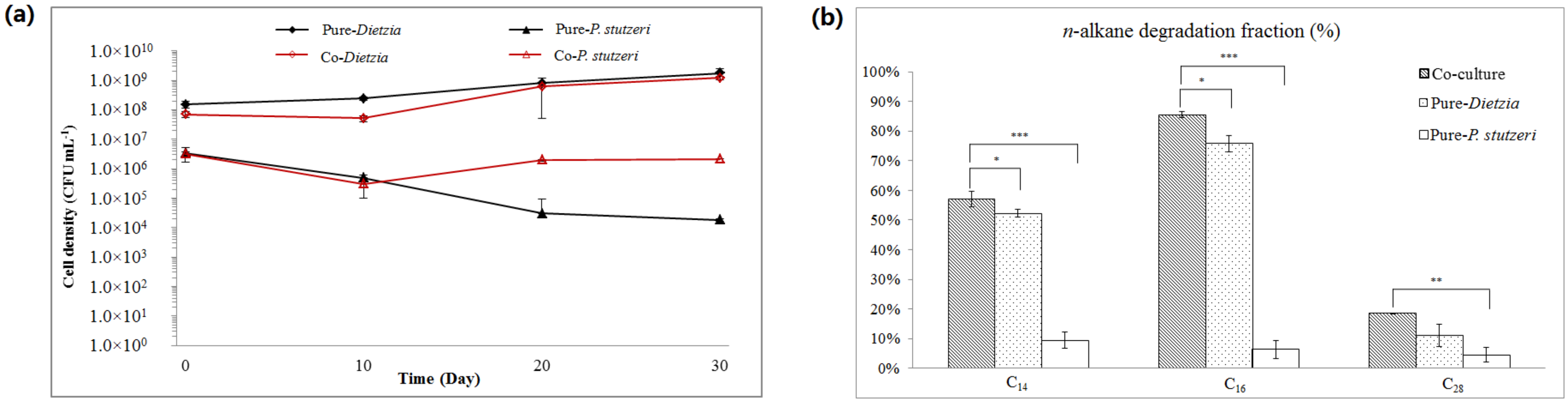

Figure S3. Time courses of *Dietzia* sp. DQ12-45-1b and *P. stutzeri* SLG510A3-8 cell densities (CFU mL^{-1}) grown on the *n*-alkane mixture separately (pure-*Dietzia* sp. and pure-*P. stutzeri*) or together (co-*Dietzia* sp. and co-*P. stutzeri*) (a); histogram showing *n*-alkane degradation fractions by the microbial community (co-culture) and *Dietzia* sp. DQ12-45-1b (pure-*Dietzia* sp.) and *P. stutzeri* SLG510A3-8 (pure-*P. stutzeri*), respectively (b). *, $p < 0.05$; **, $p < 0.01$, ***, $p < 0.005$.

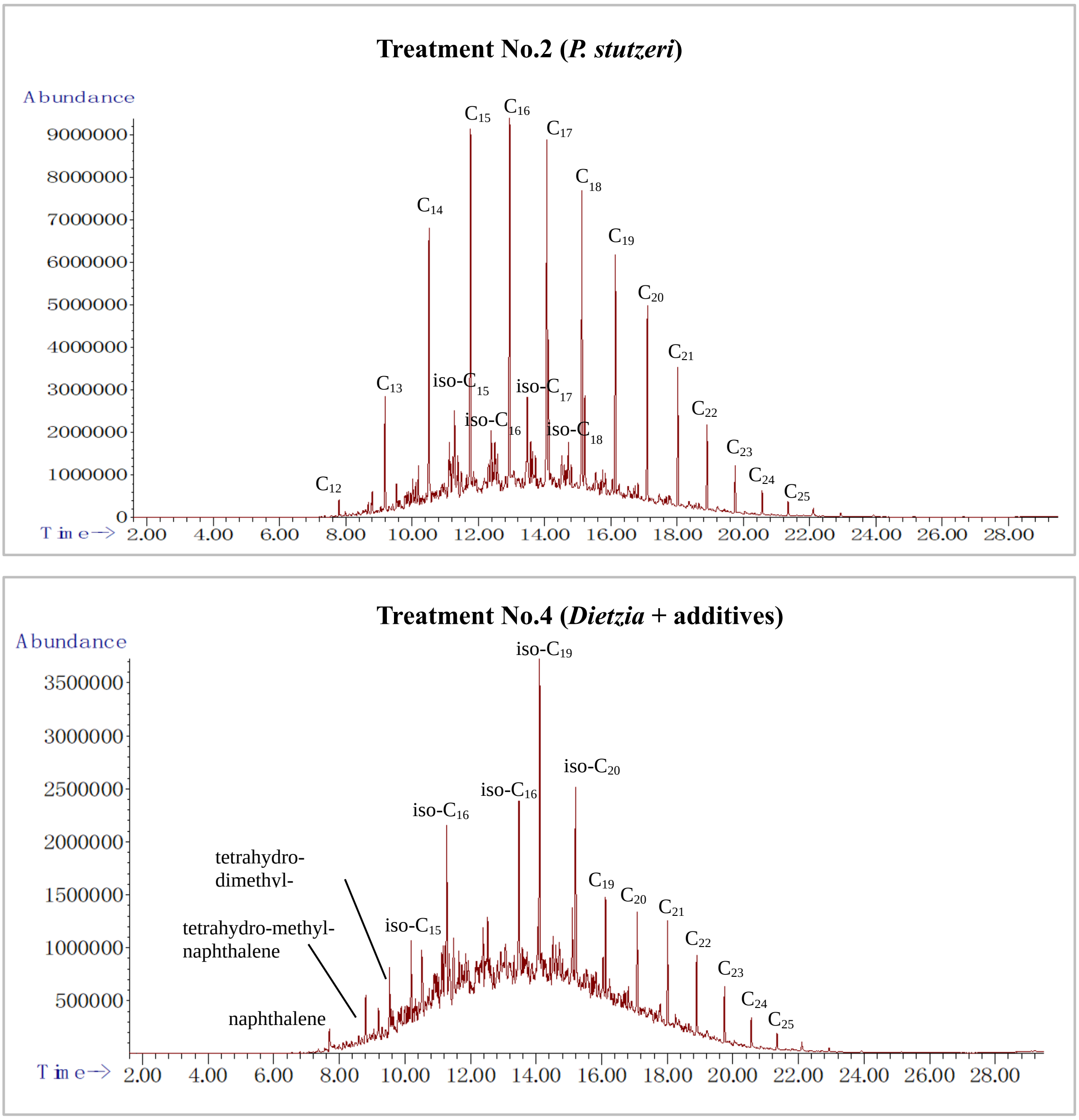

Figure S4. Hydrocarbon composition in the residual diesel oil after a 15-day disposal by using *P. stutzeri* individually (Treatment No.2) and using *Dietzia* sp. individually with the addition of slightly amounts of sodium acetate and sodium glutamate (Treatment No.4).

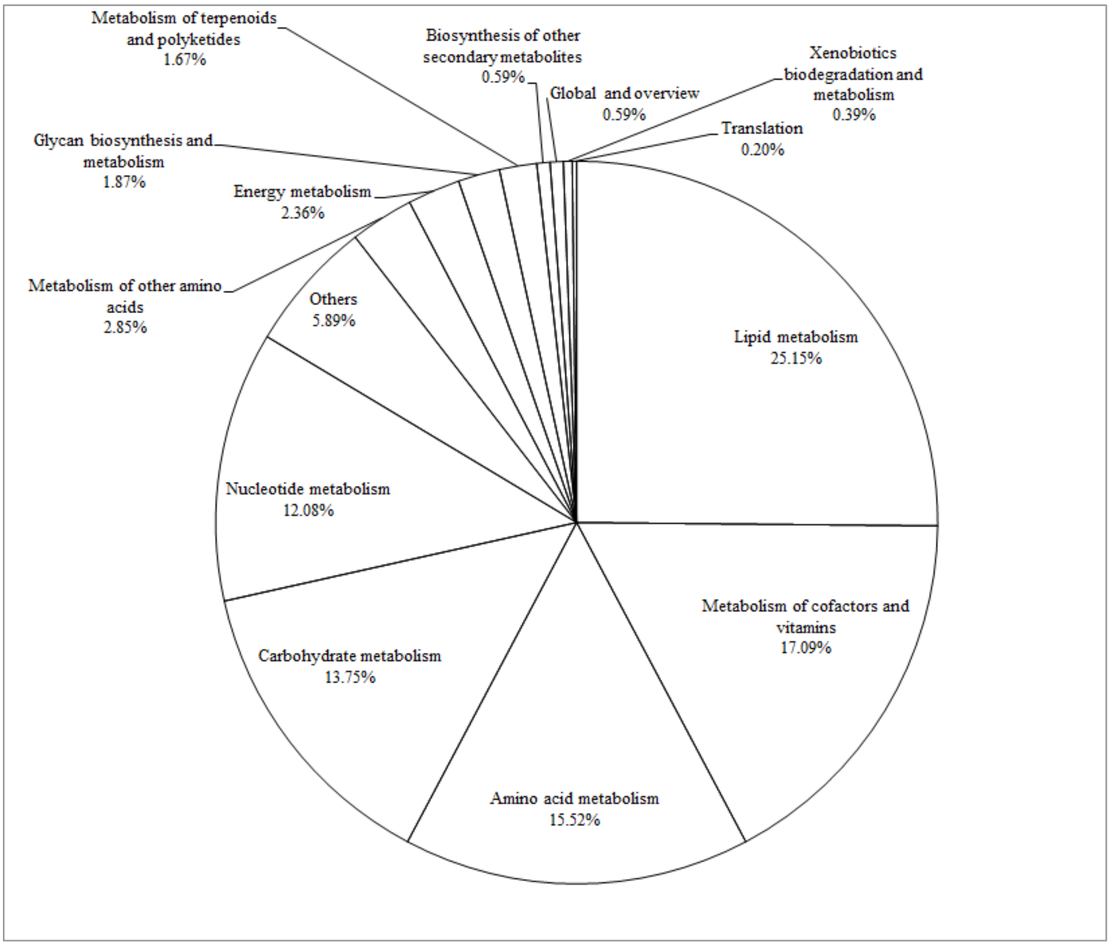

Figure S5. Assignment of the *i*BH925 metabolic reactions to the metabolic subsystems

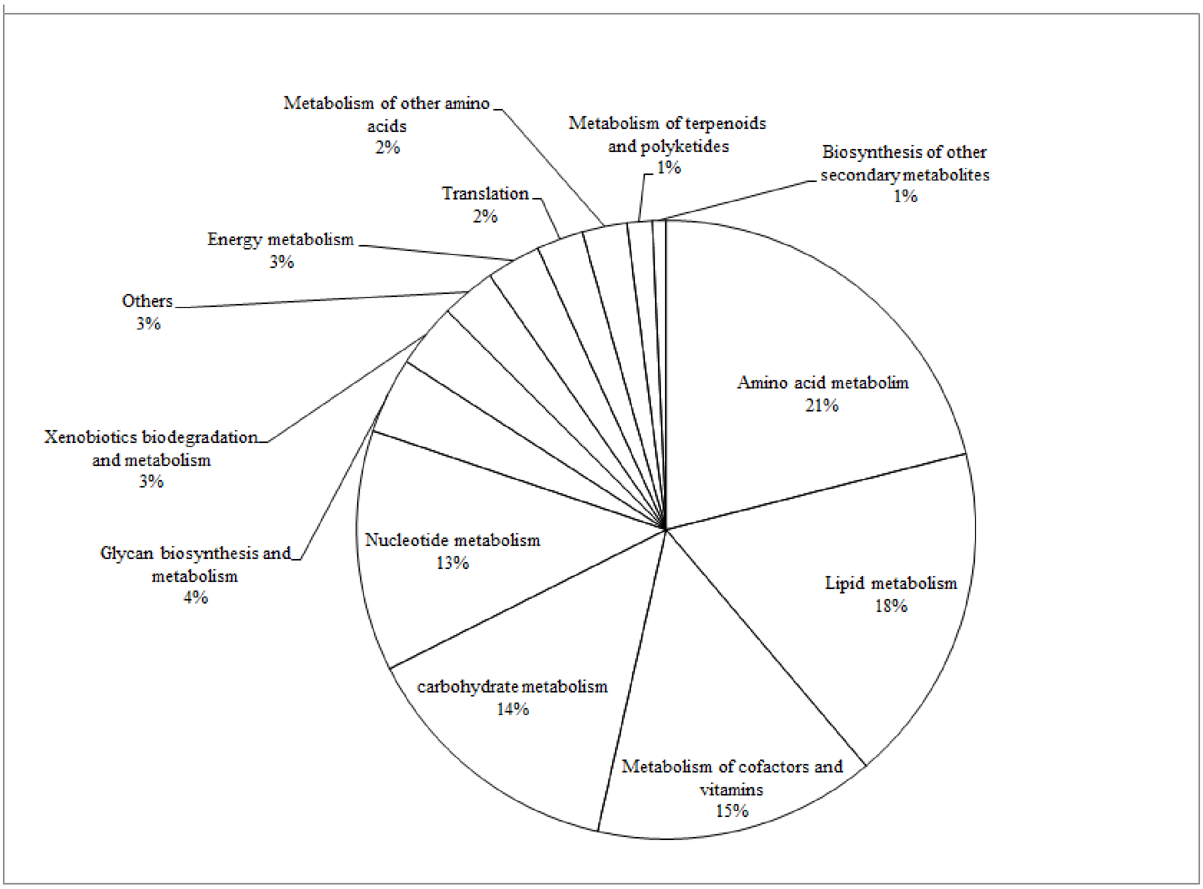

Figure S6. Assignment of the *i*BH983 metabolic reactions to the metabolic subsystems

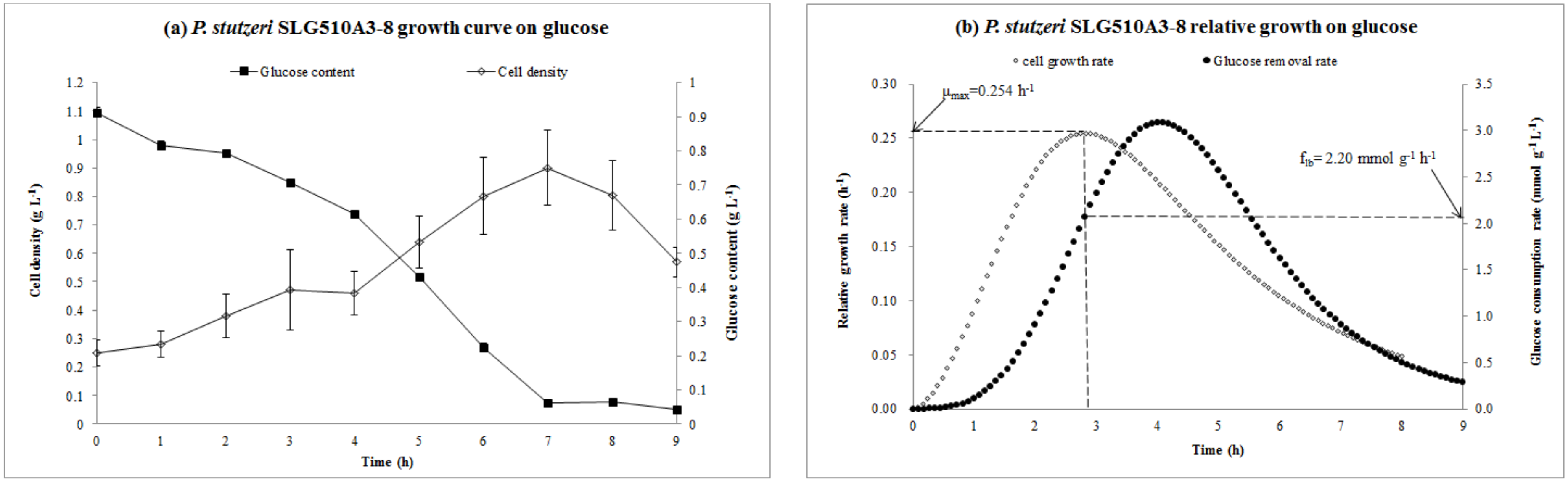

Figure S7. The time courses of *P. stutzeri* SLG510A3-8 cell density (g L^{-1}) grown on glucose and residual glucose content (g L^{-1}) (a); the time courses of the relative cell growth rate (h^{-1}) and glucose relative consumption rate (mmol g^{-1} h^{-1}) for the strain (b).

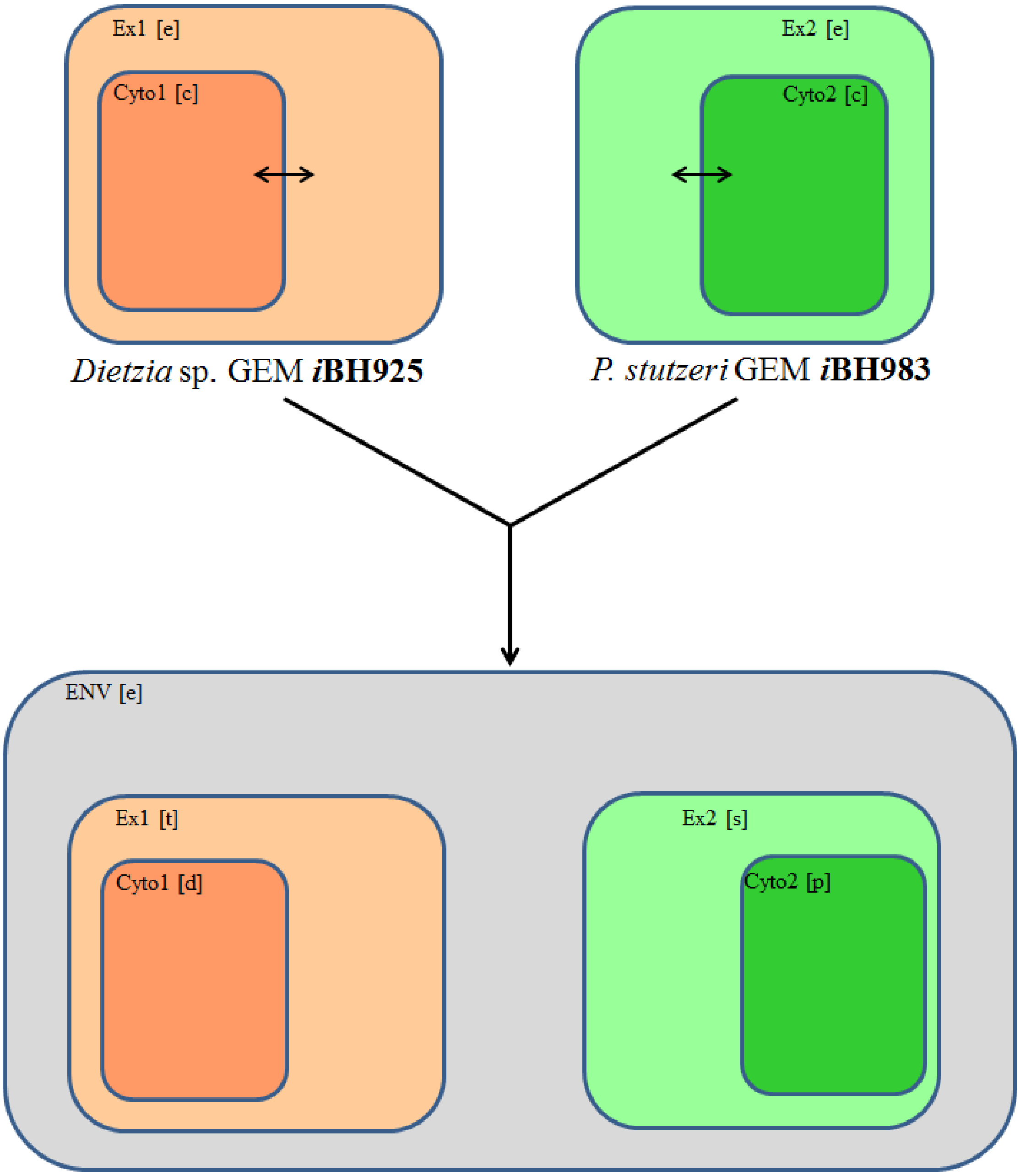

Figure S8. Scheme showing the workflow of building the two-species metabolic model. Ex1 and Cyto1 representing the extracellular space and cytoplasm of *Dietzia* sp., Ex2 and Cyto2 representing the extracellular space and cytoplasm of *P. stutzeri*, and ENV representing interspecific space shared by the two strains.

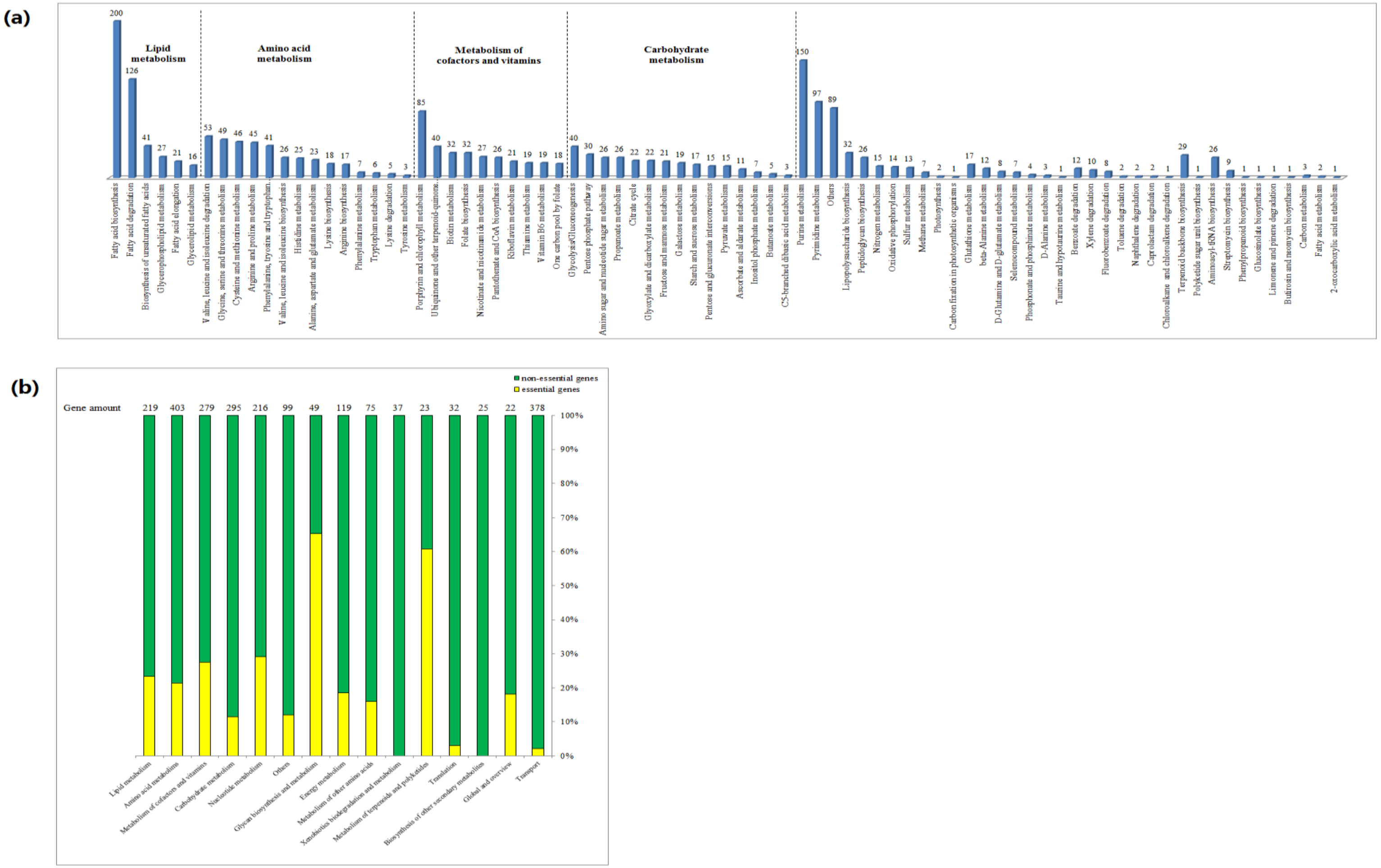

Figure S9. Histograms showing distribution of *i*BH1908 metabolic reactions in a variety of specific metabolic pathways (a) and essential gene amounts in each metabolic subsystems (b). Subsystems of high importance in (a) were highlighted in bold.
